# Entropy Analysis of COVID-19 Cardiovascular Signals

**DOI:** 10.3390/e23010087

**Published:** 2021-01-09

**Authors:** Dragana Bajić, Vlado Đajić, Branislav Milovanović

**Affiliations:** 1 Faculty of Technical Sciences, University of Novi Sad, Novi Sad 21000, Serbia; 2 Neurology Clinic, University Clinical Centre of the Republic of Srpska, 78000 Banja Luka, Bosnia and Herzegovina; vlado.djajic@kc-bl.com; 3 Faculty of Medicine, University of Belgrade, Belgrade 11000, Serbia; branislav_milovanovic@vektor.net; 4 University Hospital Center Bežanijska Kosa, Belgrade 11000, Serbia

**Keywords:** COVID-19, sample entropy, dependency structures, composite multiscale entropy, copula

## Abstract

The world has faced a coronavirus outbreak, which, in addition to lung complications, has caused other serious problems, including cardiovascular. There is still no explanation for the mechanisms of coronavirus that trigger dysfunction of the cardiac autonomic nervous system (ANS). We believe that the complex mechanisms that change the status of ANS could only be solved by advanced multidimensional analysis of many variables, obtained both from the original cardiovascular signals and from laboratory analysis and detailed patient history. The aim of this paper is to analyze different measures of entropy as potential dimensions of the multidimensional space of cardiovascular data. The measures were applied to heart rate and systolic blood pressure signals collected from 116 patients with COVID-19 and 77 healthy controls. Methods that indicate a statistically significant difference between patients with different levels of infection and healthy controls will be used for further multivariate research. As a result, it was shown that a statistically significant difference between healthy controls and patients with COVID-19 was shown by sample entropy applied to integrated transformed probability signals, common symbolic dynamics entropy, and copula parameters. Statistical significance between serious and mild patients with COVID-19 can only be achieved by cross-entropies of heart rate signals and systolic pressure. This result contributes to the hypothesis that the severity of COVID-19 disease is associated with ANS disorder and encourages further research.

## 1. Introduction

The world has faced an outbreak of a new coronavirus disease (COVID-19) at a pandemic level. It is caused by severe acute respiratory syndrome-coronavirus 2 (SARS-CoV-2), and it may initiate grave respiratory problems, so the primary research focus is on pulmonary complications.

Other problems have been reported, including cardiovascular [1,2,3], shown to be a possible contributor to the mortality associated with COVID-19 [4,5]. Still, there is no explanation of coronavirus mechanisms that trigger the changes in electrocardiogram and blood pressure waveforms, as well as the appearance of cardiac autonomic nervous system dysfunction. Despite numerous databases that collect the papers devoted to COVID-19 [6,7], we found only one case study of heart rate (HR) and its variability (HRV) time-series, performed in a single patient [8].

We believe that the complex mechanisms that alter the status of the cardiac autonomic nervous system (ANS) could be tackled only by advanced multidimensional analysis of many variables, obtained both from the source signals (ECG and blood pressure waveforms) and from laboratory analysis and detailed patient’s history. Such an analysis, based on machine-learning techniques, will increase knowledge of causative relationships and consequential effects related to the ANS disorder in COVID-19 patients. This paper aims to analyze different entropy measures as potential dimensions of the multidimensional space of cardiovascular data.

Entropy itself is an intriguing function, ever since its introduction in thermodynamics and statistical mechanics as a measure of disorder, proportional to the logarithm of the number of microscopic realizations of a macro-system [9]. The transition to telecommunications was in Shannon’s fundamental paper on the uncertainty function of transmitted signals [10], also called entropy (J. von Neumann: “You should call it entropy, for two reasons. In the first place your uncertainty function has been used in statistical mechanics under that name, so it already has a name. In the second place, and more important, nobody knows what entropy really is, so in a debate you will always have the advantage.”) [11]. The generalization of Shannon’s entropy for dynamic systems was introduced by Kolmogorov and elaborated by Sinai [12]. An easy implementation of theoretically demanding Kolmogorov–Sinai (KS) entropy was enabled by approximate and sample entropies (*ApEn* and *SampEn*) [13,14], immediately recognized within the research society [15]. Since then, numerous entropy variations of Shannon and KS entropies have been used as analytical tools in almost all scientific disciplines.

This paper analyzes systolic blood pressure (SBP) time series, and series of intervals between the successive R peaks of electrocardiogram (RR intervals, or RRI) of 116 COVID-19 patients and 77 healthy volunteers, applying, besides classical entropies, composite multiscale entropy [16,17] of dependency time series [18], copula analysis [19] for the entropy of probability integral transformed signals [20], and entropy of binary differentially coded signal expressed as the entropy of joint symbolic dynamics [21] and as the binarized entropy [22]. Thus, the signal processing tools are known, but to our knowledge, such a cardiovascular database recorded from COVID-19 patients has not been analyzed yet, and certainly not by entropy. Based on the results, we can single out the parameters most relevant for multivariable analysis of cardiovascular patient’s status.

## 2. Materials and Methods

### 2.1. Signal Acquisition

**Figure 1:**
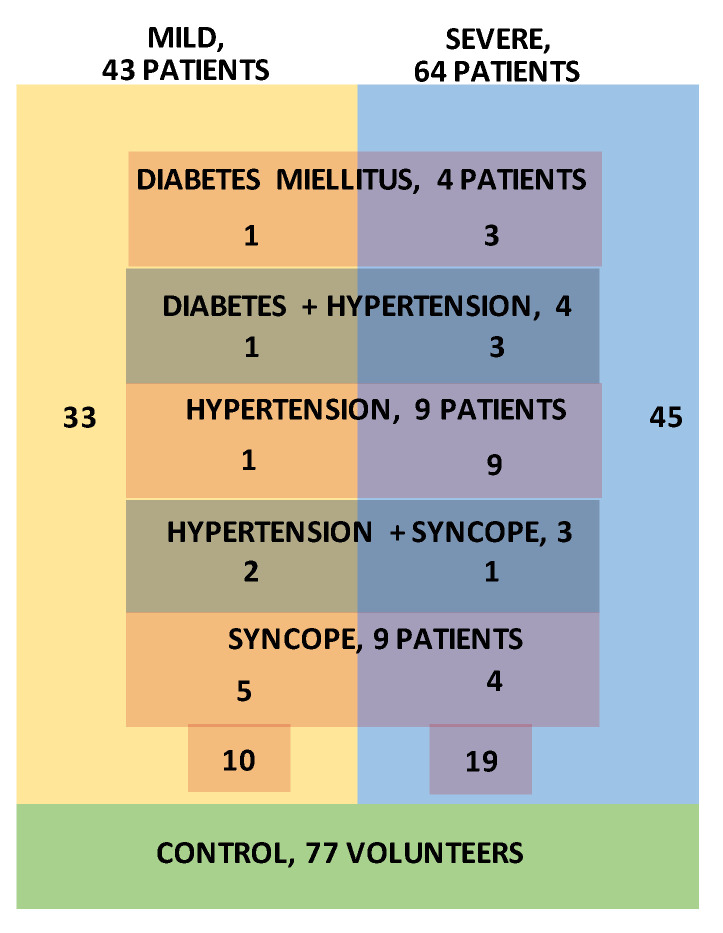
Subject grouping. Severe group: 64 patients, without associate diseases: 45 patients. Mild group: 43 patients, without associate diseases: 33 patients. Diabetes: 4 patients (1 mild and 3 severe), diabetes + hypertension: 4 patients (1 + 3), hypertension: 9 patients (1 + 8), hypertension + syncope: 3 patients (2 + 1), and syncope: 9 patients (5 + 4).

Signal acquisition was performed at the University Clinical Centre of the Republic of Srpska, Bosnia, and Herzegovina, using a TaskForce^®^ monitor [23] that records electrocardiogram (ECG) signals and blood pressure (BP) waveforms with the sampling frequency of 1000 Hz. The device simultaneously extracts beat-to-beat SBP and RRI time series. The COVID-19 diagnosis was confirmed by PCR nasopharyngeal test and serology test for specific COVID-19 IgG and IgM antibodies. Out of 116 recorded SBP-RRI time series, visual inspection excluded 9 pairs, either due to insufficient length or because of obvious recording problems. The remaining patients were separated into a severe group—patients with interstitial pneumonia confirmed by chest radiography, 64 patients, and mild group, 43 patients. Further subgroups were formed from patients with previous associate diseases: Diabetes mellitus (4 patients), hypertension (9 patients), syncope (9 patients), diabetes mellitus + hypertension (4 patients), and hypertension + syncope (3 patients). The patient distribution is presented in Figure 1. It should be noted that the number of patients with associated diseases was too small to provide statistically significant results, so they are presented as an illustration. The research follows the Helsinki Declaration and it is approved by the Ethics Committee of University Clinical Centre of the Republic of Srpska, Bosnia, and Herzegovina, No. 01-5617/3-20 from May 2020.

Signal acquisition from healthy volunteers (controls) was performed at University Hospital Center Bežanijska Kosa, Belgrade, Serbia, also using a TaskForce^®^ monitor. To ensure corona-free conditions, for this study we used signals recorded three years before the corona outburst. Signals were recorded from 77 medically checked controls, age and sex-matched to patients. The research follows the Helsinki Declaration and it is approved by the Ethics Committee of University Hospital Center Bežanijska Kosa, Belgrade, Serbia, No. 11754/3 from December 2015.

The list of acronyms is given in Table 1.

### 2.2. Signal Preprocessing

**Figure 2:**
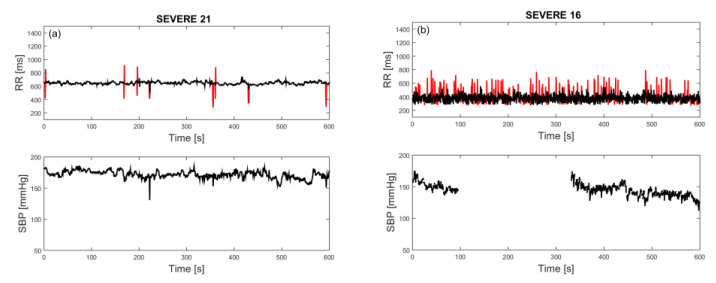
Artifacts in SBP and RRI time series. (**a**) Moderate artifacts in RRI and SBP without the artifacts, patient #21; (**b**) turbulent RRI, characteristic of severe group of patients, and SBP with large interrupt, patient #16. Red lines: Removed artifacts; black lines: Corrected signal.

After the first visual examination, RRI time series were filtered using an adaptive filter developed particularly for this type of signal [24] and replotted. Most of the time series had a low amount of artifacts. A time series with a moderate amount of artifacts, but still below 2%, is presented in Figure 2a. However, within the severe group of patients, more than 25% had RR intervals that could be described as “turbulent” (Figure 2b), where filtering could not induce any improvement. Since such signals are a characteristic of the severe group of patients, as shown in medical analysis with Poincaré plots [25], they remained in our study.

The SBP time series suffer from occasional interruptions. Missing samples were mostly solitary, infrequent, and easily amended by the mean value of neighboring samples. However, there were eight SBP time series (two ones in the mild group and six ones in the severe group) where a considerable amount of the samples was missing, either contiguous or distributed (occurrence of both is visible in Figure 2b, lower panel). For these cases, SBP and joint SBP–RRI analyses were not performed.

Some of the analyses require stationary signals. In such cases, a very low-frequency component (trend) is removed by a filter designed for biomedical time series [26]. For joint analyses of SBP and RRI time series, we performed standard score (z-score) centralizing and normalizing the time series.

The minimum sample size was determined for a 95% confidence level, with a tolerable error level of 0.05, for all estimated mean values and standard deviations [27]. The worst case was 31 subjects. This condition was achieved for Mild, Severe, and Control groups, but not for patients with associated diseases. The latter results are presented to provide a complete insight into the signals of patients with COVID-19.

The figures presenting SBP and RRI time series for all subjects are attached in the Supplementary Materials (184 signal pairs).

### 2.3. Analytical Tools

The analytical tools are explained in brief, as the detailed explanation can be found in numerous journal papers, books, and tutorials; just a small portion is quoted in the text.

#### 2.3.1. Parametric Methods: Sample Entropy and Its Variations

Among the parametric methods, approximate entropy is more similar to KS entropy [13], but we implemented sample entropy (*SampEn*) as more robust, insensitive to time series length (except for short time series), and symmetric when applied to a pair of signals [14]. *SampEn* estimates the complexity of the time series based on two parameters: Embedded dimension *m* and threshold (tolerance) *r.* The estimation is based on “template matching”, where the time series x∈{SBP,RRI} of length *N* is divided into *N − m* overlapping vectors $X_{m}\left(i\right)$ of length *m*, $X_{m}\left(i\right)=\left[x_{i}\;x_{i+\tau }\cdots x_{i+\left(m-1\right)\cdot \tau }\right]$. The $\left(\begin{array}{c} N-m-1\\ 2 \end{array}\right)$ vector pairs $X_{m}\left(i\right),\;\;X_{m}\left(j\right),\;i,j=1,\cdots ,N-m,\;i\neq j$ are compared to each other to find matched (similar) ones, where the similarity criterion is their maximal absolute distance that should be below the threshold *r*:

(1) $$dist\left(X_{m}\left(i\right),X_{m}\left(j\right),\right)=\;\underset{k=0:m-1}{\max}\left|x_{i+k\cdot \tau }-x_{j+k\cdot \tau }\right|$$

The procedure is repeated for an increased embedded dimension (*m* + 1). Then the numbers of matchings for *m* + 1 and *m* are divided, and the negative logarithm of the quotient yields the simple equation of *SampEn*:

(2) $$SampEn\left(N,m,r,\tau \right)=-\log\left(\sum_{i=1}^{N-m}\sum_{j=1,j\neq i}^{N-m}\frac{I\left\{dist\left(X_{m+1}\left(i\right),X_{m+1}\left(j\right),\right)\;<\;r\right\}}{I\left\{dist\left(X_{m}\left(i\right),X_{m}\left(j\right),\right)\;<\;r\right\}}\right)$$

In this equation, *τ* is a time delay between the samples of the original series *x*. The vectors are usually contiguous, so *τ* is, as a rule, set to one [28]. I{ } is the indicator function equal to 1 if the condition within the curly brackets is fulfilled. In this case, the condition is the similarity of vectors $X_{m}\left(i\right)\;\mathrm{and}\;X_{m}\left(j\right)$, so the summation of the indicator function counts the similar vectors.

Small *SampEn* corresponds to self-similar time series, as the number of matches does not decrease significantly with an increase of vector length. In this study, we used embedded dimension *m* = 2, and threshold *r* = 0.3 that additionally stabilizes the estimation [29]. We estimated *SampEn* from RRI and SBP time series and *Cross-SampEn* (*XSampEn*) from joint RRI–SBP time series. The procedure of *XSampEn* is similar to *SampEn*, except that the similarity is tested comparing the vectors from different time series. We also applied *SampEn* to signals transformed by probability integral transform (PIT). PIT eliminates the influence of amplitude distribution, as the distribution of the resulting signals is uniform [20,30].

The function that transforms signal x∈{SBP,RRI} into a new signal *u* uniformly distributed on segment [0,1] is cumulative distribution function $F_{x}\left(x\right)$ of the signal *x* itself, $u\;=F_{x}\left(x\right)$. In our case, the signal amplitudes are bounded by physiological limitations, $x_{min}\leq x\leq x_{max}$, so $F_{x}\left(x\right)$ can be defined as:

(3) $$F_{x}\left(x\right)=\{\begin{array}{c} 0,\;x<x_{min}\\ F_{x}\left(x\right),\;x_{max}\leq x\leq x_{min}.\\ 1,x>x_{max} \end{array}$$

For a fixed $x_{0}$, $F_{x}\left(x_{0}\right)=\mathrm{Pr}\left\{x\leq x_{0}\right\}$ by definition, where “Pr” stands for probability. If $u_{0}=F_{x}\left(x_{0}\right)$, then $F_{x}\left(x_{min}\right)=\;0\leq u_{0}\leq F_{x}\left(x_{max}\right)=1$. As $x_{0}=F_{x}^{-1}\left(u_{0}\right)$, then $\mathrm{Pr}\left\{u\leq u_{0}\right\}=\;F_{u}\left(u_{0}\right)=\mathrm{Pr}\left\{x\leq x_{0}\right\}=\;\mathrm{Pr}\left\{x\leq F_{x}^{-1}\left(u_{0}\right)\right\},$ which is, according to the definition, $F_{x}\left(F_{x}^{-1}\left(u_{0}\right)\;\right)=u_{0}$. So the cumulative distribution function after the PI-transform is equal to:

(4) $$F_{u}\left(u_{0}\right)=\{\begin{array}{c} 0,\;u_{0}<0\\ u_{0},\;0\leq u_{0}\leq 1\\ 1,u_{0}\geq 1 \end{array}.$$

Its derivate defines the probability density function (dropping the index “0”):

(5) $$f_{u}\left(u\right)=\{\begin{array}{c} 0,\;u<0\\ \frac{du}{du}=1,\;0\leq u\leq 1\\ 0,u>1 \end{array}.$$

So, the probability distribution function of PI-transformed signal is uniform on segment [0,1].

The joint probability density function (PDF) of PIT signals is copula density. Contrary to classical PDF that presents a two-dimensional amplitude distribution in the SBP–RRI plane, copula density presents the density of PI-transformed SBP and RRI. As each of the PI-transformed signals is defined on [0,1] interval, copula density is defined on a two-dimensional [0,1]^2^ plane known as copula density plane. The copula density shows the dependency structure of SBP and PI signals, as its intensity corresponds to the level of their coupling. If copula type is carefully chosen, dependency quantification has better sensitivity if compared to classical Kendal’s, Spearman’s, and Pearson’s tests [27]. It was shown [20] that Frank’s copula is well suited for the SBP–RRI relationship:

(6) $$C^{\left(F\right)}\left(u_{1},\ldots ,u_{D}\right)=-\theta^{-1}\cdot \log\left[1+\frac{\prod_{i=1}^{D}\left(e^{-\theta \cdot u_{i}}-1\right)}{{\left(e^{-\theta }-1\right)}^{D-1}}\right],\;\theta \epsilon [-\infty ,\infty )/\left\{0\right\}.$$

This expression presents a theoretical Frank’s copula $C^{\left(F\right)}$, where *D* is the number of signals (in our SBP–RRI space, the number of dimensions is *D* = 2), $u_{1},\ldots ,u_{D}$ are probability integral transformed signals (i.e., uniform signals), and *θ* is the level of signal coupling.

A point in the copula density plane shows the level of dependency between the corresponding SBP and RRI values. So, if the coordinates of copula density are transformed back to real SBP-PI plane by inverse PI-transformation, the dependency level expressed by copula will be associated to a real SBP-PI pair positioned along the time axis. In such a way a new time series that show the fluctuations of signal coupling level can be created [18].

For analysis of this new time series, we have applied composite multiscale entropy, as it provides insight into the complexity of fluctuation signal (coupling level) over a range of time scales, to find if there exists a scale that would be the most informative. Multiscale entropy (MSE) and its composite improvement (CMSE) yield an entropy for different scale values *S*. For each scale, samples of a new time series are formed as non-overlapping averages of *S* adjacent original samples. For CMSE, *S* different signals per scale are formed, one for each shift within the scale. The estimated entropies are averaged. For MSE, only the first shift is taken into account. The number of samples in time series decreases to ⌊*N/S*⌋, where ⌊*∙*⌋ denotes lower integer value (floor) of *N/S*.

Samples $x_{MS}^{\left(S\right)}\left(i\right)$, and $x_{CMS}^{\left(S\right)}\left(i,k\right),\;$as well as MSE and CMSE are formed as:

(7) $$x_{MS}^{\left(S\right)}\left(i\right)=\frac{1}{S}\cdot \sum_{j=\left(i-1\right)\cdot S+1}^{i\cdot S}x_{j},\;i=1,\ldots ,\lfloor N/S\rfloor \;;\;MSE\left(S\right)=SampEn\left(x_{MS}^{\left(S\right)}\right);\;\;$$

$$x_{CMS}^{\left(S\right)}\left(i,k\right)=\overset{i\cdot S+k}{\underset{j=\left(i-1\right)\cdot S+1+k}{\sum }}x_{j},\;i=1,\ldots ,\lfloor N/S\rfloor ;\;k=0,\;\ldots ,S-1;$$

(8) $$CMSE\left(S\right)=SampEn\left(x_{CMS}^{\left(S\right)}\right)\;=\frac{1}{S}\cdot \sum_{k=0}^{S-1}SampEn\left(x_{CMS}^{\left(S\right)}\left(k\right)\right).\;\;$$

#### 2.3.2. Entropy of Binary Differentially Coded Signals

Binary differential coding is a coarse method, preserving only information whether the signal amplitude rises or falls, without the information of its value. We applied two tools: Binarized entropy [22], and entropy of joint symbolic dynamic [21], known as Shannon JSD and denoted JSD_Sh_. Both methods take into account the temporal dynamics of signals, and both methods start with binary differential coding.

Signal x∈{SBP,RRI} is binary differentially coded as follows:

(9) $$b_{i}=\{\begin{array}{c} 0,x_{i+1}-x_{i}\leq 0\\ 1,x_{i+1}-x_{i}>0 \end{array}\;,\;i=1,\;\cdots ,\;N-1.$$

The negative increment can induce bias in RRI time series, irrelevant for a sampling frequency of 1000 Hz. It can be eliminated if the source signals (ECG or blood pressure waveforms) were filtered by an ideal low pass filter with resolution Δt=0.1 ms or less. An artificial but effective way to eliminate the bias when $\;x_{i+1}-x_{i}=0\;$is to generate a random number with uniform distribution, and then to associate 0 to binary sample $b_{i}\;$if the generated random number is less than or equal to 0.5, or to associate 1 if it is greater than 0.5.

Binarized entropy (*BinEn*) operates similarly to *SampEn*. The difference is a distance measure that is Hamming’s, and threshold *r* that is discrete and can take any value from 0 to *m* − 1. It should be noted that, if *BinEn* is defined according to principles of *ApEn*, then the threshold set to *r* = 0 yields the classical Shannon’s entropy for binary sources.

Joint symbolic dynamic (JSD) is formally different from other entropy measures, as it does not implement sample entropy, but Shannon’s entropy. It is included all the same as (a) it operates over adjacent samples, so it includes dynamics, as suggested by its name, and (b) it also includes binary differential coding.

Having obtained binary series *b* = [*b*_1_ *b*_2_ … *b_N_*_−1_], *m* adjacent non-overlapping bits form the symbols of SBP time series:

(10) $$s_{j}^{SBP}=\;\sum_{k=1}^{m}b_{\left(j-1\right)\cdot m+k}\cdot 2^{k-1},\;\;\;j=1,\;2,\;\ldots ,\;\lfloor \left(N-1\right)/m\rfloor .$$

The procedure is repeated for the second time series (here RRI), so the final joint symbol $S_{j}\;$is two dimensional:

(11) $$S_{j}=s_{j}^{SBP}s_{j+L}^{RRI},\;j=1,\;2,\;\ldots ,\;\lfloor \left(N-1-L\right)/m\rfloor .$$

where *L* denotes a time lag between SBP and RRI time series. Then classical Shannon entropy estimation can be applied.

#### 2.3.3. Control Signals and Results Presentation

We used two types of pseudo-random control signals to test the upper bounds of estimated values: Artificially generated Gaussian signals, and isodistributional surrogate signals. They estimate limiting values of the applied method if the exact values, calculated by formula, are unavailable. The Gaussian signal comprises statistically independent and identically distributed Gaussian samples for which Shannon entropy is maximal. Isodistribution surrogates enable estimation of limiting values for each patient separately: The original signal samples are randomly permuted, preserving the amplitude distribution, but destroying statistical dependence of the samples [31]. For each patient’s signal, 50 surrogate signals are generated, and the results estimated from them are averaged.

The logarithms that are used for evaluations are natural. If not stated differently, all the results are presented as the mean ± standard error of the mean (SE).

## 3. Results and Discussion

The basic parameters of patients and controls—heart rate and systolic blood pressure—are presented in Table 2. It is, however, obvious that the mean value of systolic blood pressure either remained the same (Severe group) or decreased, though the decrease was not significant in a statistical sense. Even the patients that already had hypertension prior to COVID-19 infection had low mean SBP values (hypertension, and hypertension + syncope groups). The only exception was two patients with SBP that exceeded 150 mmHg and altered the statistics of the diabetes + hypertension group. The overall conclusion is that influence of COVID-19 on cardiac ANS dysfunction cannot be elucidated using classical cardiac parameters. There are no significant differences between COVID-19 patients and healthy controls, except that patients with hypertension could get lower values of systolic blood pressure than usual.

**Figure 3:**
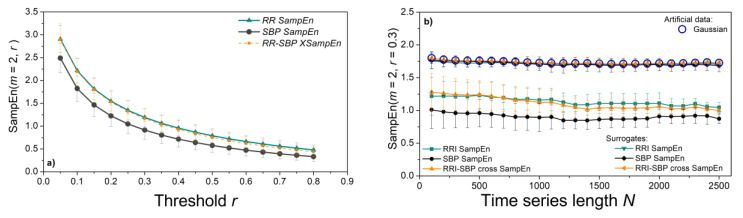
*SampEn* profiles of SBP, RRI, and cross-SBP-RRI time series in control group. (**a**) Threshold profile; (**b**) length profile, including artificial Gaussian time series and isodistributional surrogates of SBP, RRI, and cross-SBP-RRI time series. Graphs are presented as mean ± SE (standard error of mean).

To explore the values of *SampEn* measures, we plotted entropy profiles. The threshold profile in Figure 3a estimates *SampEn* from RRI and SBP time series for a range of different thresholds, keeping the length of time series fixed (*N* = 800); length profile in Figure 3b) keeps the threshold fixed (*r* = 0.3) and plots *SampEn* for a range of series lengths. Entropy estimates monotonously decrease with the threshold, while the length of time-series does not influence the estimates. This result is important for the multiscale entropy applications. We kept the same time series length (*N* = 1000) for all experiments, except for an illustrative example of patients with associated diseases who have shorter time series. The entropy of the surrogate time series is high (1.77) and overlaps with the results of the Gaussian time series, a characteristic of statistically independent data (Figure 3b).

**Figure 4:**
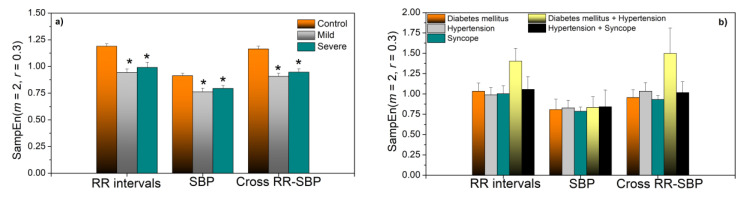
*SampEn* of SBP, RRI, and cross-SBP-RRI time series. (**a**) Control, Mild, and Severe groups; (**b**) patients with associate diseases. * Statistically significant results are assessed for *p* ≤ 0.05 (Mann–Whitney test).

**Figure 5:**
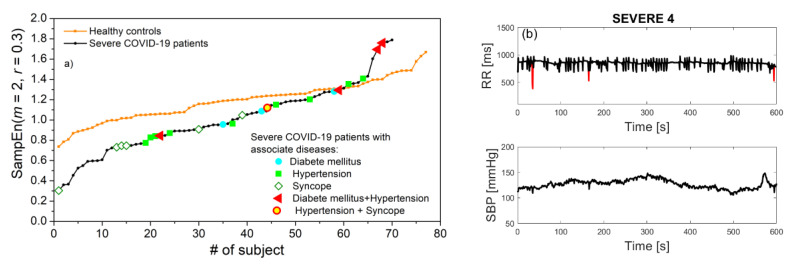
(**a**) *SampEn* of RRI in ascending order for control and Severe groups. (**b**) RRI and SBP time series of signal with ectopic beats; the *SampEn* of this signal was very low.

The statistics of *SampEn* are presented in Figure 4a. Estimates in Mild and Severe COVID-19 patients are almost the same, but significantly decreased with respect to the control group. Statistically significant results are assessed for *p* ≤ 0.05 (Mann–Whitney test). Figure 4b) presents the results of patients with associate diseases presented for illustrative purposes. To assess the extent to which the entropy of COVID-19 patients is indeed lower than the entropy of healthy volunteers, in Figure 5a we present entropy estimates of RRI series in ascending order for Control and Severe groups. The severe patients with associate diseases are also included. We can observe that most of the COVID-19 patients have *SampEn* below the control group, but some of them have values that approach the maximal values of statistically independent data.

The number of patients with associated diseases is too small for drawing conclusions, but it seems that patients with syncope are shifted towards low entropy values, and patients with both diabetes and hypertension have high entropy. It turns out that RRI signals that we characterized as “turbulent” have either high entropy values, as signal already presented in Figure 2b, or very low value, as regularity increases in a case of repeated ectopic beats, Figure 5b.

**Figure 6:**
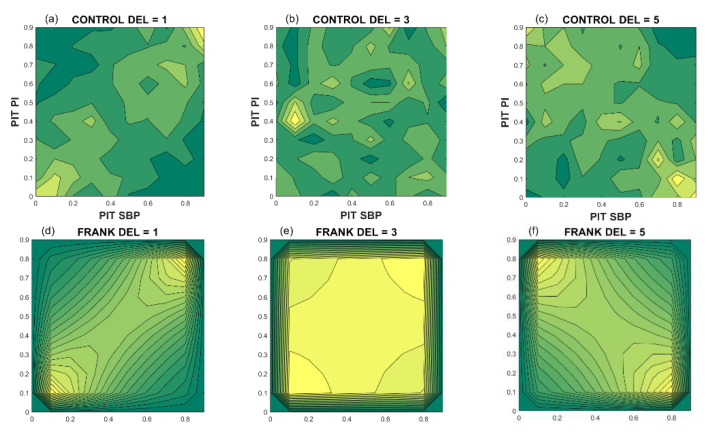
Copula density of a patient for different RRI delays with respect to SBP. Upper panels: Empiric copula density (dependency density); lower panels: Corresponding theoretical Frank copula density; (**a**,**d**): Delay is equal to 1 beat, copula parameter *θ* = 3.507; (**b**,**e**): Delay = 3 beats, *θ* = 0.261; (**c**,**f**): Delay = 5 beats, *θ* = −2.298.

To form a signal that shows the temporal fluctuations of coupling strength (dependency level) of SBP and RRI signals, we needed to perform PI transformation and to estimate copula density. Figure 6 shows the copula density (dependency structures) of a healthy volunteer. Upper panels present empirical density for different delays of RRI signal with respect to SBP. Lower panels present the theoretical Frank copulas for copula parameters estimated from the empiric copula. For a delay equal to 1 beat, the relation between the SBP and RRI is positive, but for a delay equal to five beats, an increase of SBP induces a decrease of RRI.

**Figure 7:**
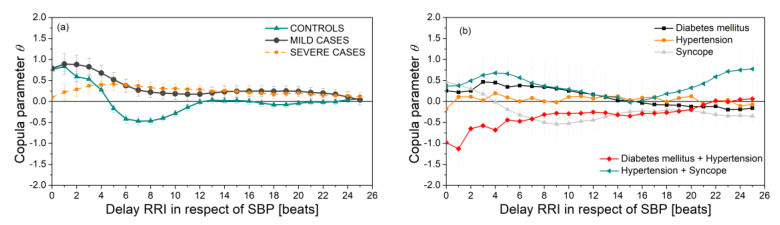
SBP-RRI dependency level (copula parameter) for different delays (in beats) of RRI with respect to SBP (in beats). (**a**) Control, Mild, and Severe groups; (**b**) groups with associate diseases.

Figure 7a presents the copula parameter (dependency level) of SBP and RRI time series as a function of delay of RRI with respect to SBP. It is notable that the results of the Severe and Mild groups are significantly different than the results of the control group. The groups with associate diseases (Figure 7b) show that the SBP–RRI relationship is almost non-existent in the hypertension group, and negative for the diabetes + hypertension group. However, the number of these patients is, as already said, too small for reliable conclusions.

**Figure 8:**
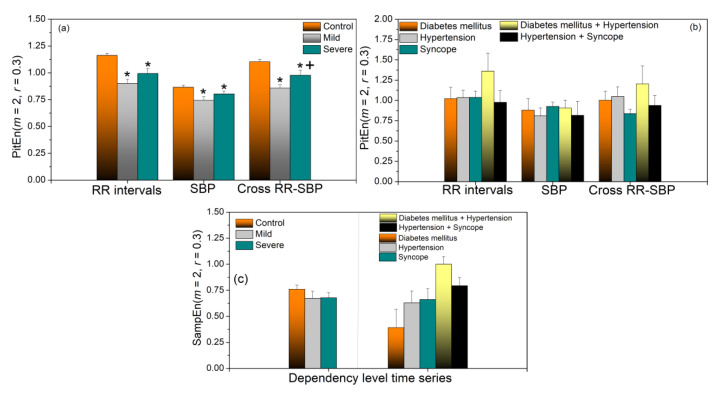
Panels (**a**,**b**): Entropy and cross-entropy of the PI-transformed time series (PIT entropy); panel (**c**): Entropy of the density level time series. Significance level *p* < 0.05, ‘*’-Patients vs. Control, ‘+’ Severe vs. Mild.

*SampEn* of the PI transformed SBP and RRI signals are presented in Figure 8a,b. These results are similar to the results of the ordinary *SampEn* shown in Figure 4, the only difference is a statistically significant distinction between the Mild and Severe groups for cross-entropy. *SampEn* of fluctuations of SBP and RRI coupling strength is presented in Figure 8c.

**Figure 9:**
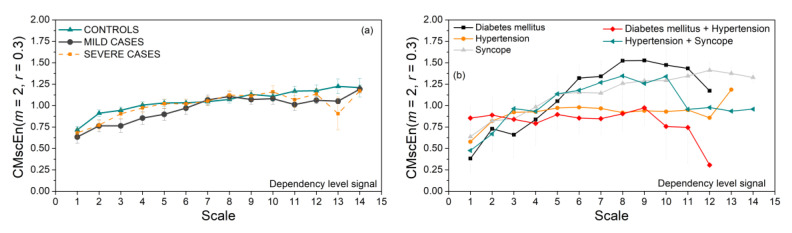
Composite multiscale entropy of PI-transformed time series; (**a**) control, Mild, and Severe groups; (**b**) groups with associate diseases.

Coupling strength of SBP and RRI signals, i.e., their dependency level time series, is used as an input for composite multiscale entropy. The results are presented in Figure 9. There are no significant differences between the control, Mild, and Severe groups (Figure 9a). Some groups of patients with associate diseases show an unexpected drop of CMSC at higher scales. The reason might be time series length *N*, which is too short and cannot reach higher time scales, so an already small number of signals decreases even further.

**Figure 10:**
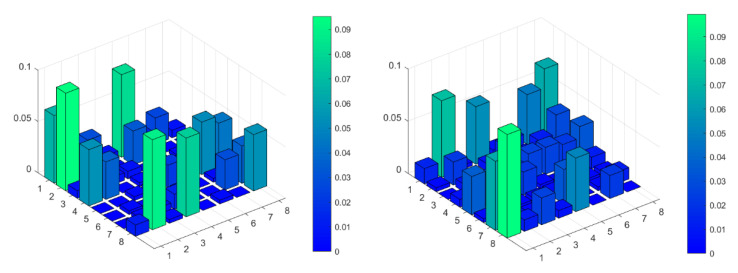
Joint symbolic dynamic entropy of same SBP and RRI signals shown in Figure 4. (**a**) Delay of RRI is equal to one beat; (**b**) delay is equal to five beats. Note the change of the position of the most frequent symbols.

**Figure 11:**
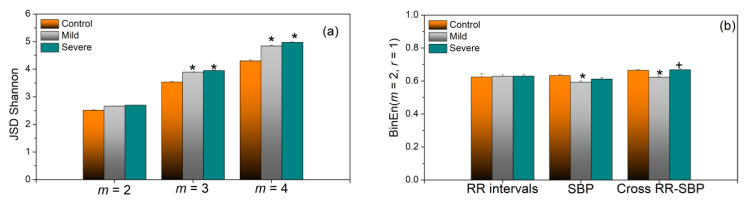
(**a**) Joint symbolic dynamic entropy; (**b**) binarized entropy; both methods are insensitive to artifacts—the values estimated from row signals and after the artifact correction differs less than 1.5%, even for the “turbulent” signals from the Severe group of patients. Significance level *p* < 0.05, ‘*’-Patients vs. Control, ‘+’ Severe vs. Mild.

The next two figures are devoted to binary differentially coded signals. Figure 10, as an illustrative example, gives standard presentation of joint symbolic dynamic of signal already presented in Figure RRI delayed by one, and by five beats with respect to SBP. Figure 11a) presents JSD_Sh_. The variance of the obtained results is very low, and the difference between COVID-19 patients (both groups) and healthy controls is significant.

Quite different results can be found in Figure 11b. Binarized *SampEn* does not make too many distinctions between the different groups. Actually, binarized entropy is robust itself, and it was always used as the binary variant of approximate entropy (*ApEn*). In this study, we consistently used *SampEn*, so we used a *SampEn*-based *BinEn* as well. Combining two very robust methods, *BinEn* and *SampEn*, created an inert method, insensitive to subtle changes.

The parameter that would not be included in our multidimensional analysis of COVID-19 data is multiscale entropy, as the estimated parameters are almost the same for all groups.

**Figure 12:**
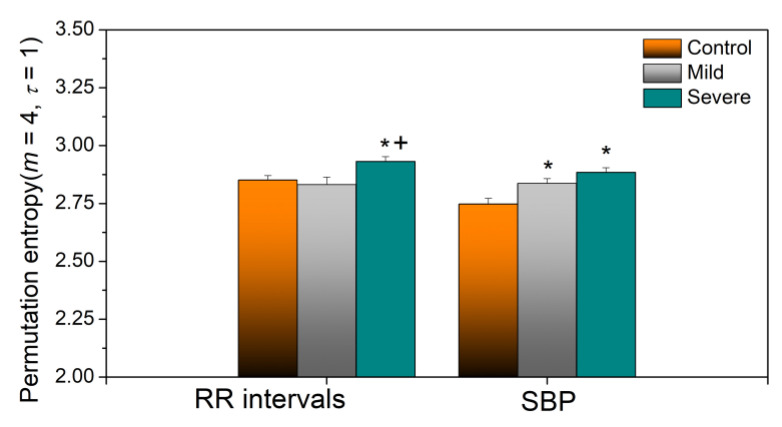
Permutation entropy for *m* = 4 and contiguous samples. *τ* = 1. Significance level *p* < 0.05, ‘*’-Patients vs. Control, ‘+’ Severe vs. Mild.

Yet another robust entropy estimate is permutation entropy, a time series tool which quantifies the complexity of a dynamic system by capturing the order relations between values of a time series and extracting a probability distribution of the ordinal patterns [32]. The results of permutation entropy analysis are presented in Figure 12.

If we compare the applied methods, it can be seen that dynamic sample entropy consistently shows higher values in control subjects than in COVID-19 patients. For *SampEn* and PitEn (Figure 4a and Figure 8a), this difference is statistically significant for the entropy of all three types of signals—RRI, SBP, and RRI–SBP. For *BinEn* (Figure 11b), the difference is significant for SBP and RRI–SBP, an interesting result because SBP signals are usually less variable than the RRI signals. The entropy of dependency time series (Figure 8c), where each sample corresponds to the instantaneous SBP–RRI dependency level, also shows an increase for healthy controls, though without the statistical significance. *SampEn*-based composite multiscale entropy also shows increased values for control subjects for almost all scales, but, again, without the significance (Figure 9a). It should be noted that the level of dependency (Figure 7a) for COVID-19 patients shows positive values for all delays of RRI with respect to SBP, without the relaxation that is visible for controls.

On the other hand, static Shannon entropy of JSD decreases in healthy controls, as if the joint symbolic structures remain firm in healthy subjects and variable in both types of COVID patients.

Considering the patients with associate diseases, due to the small sample size, nothing can be concluded. However, an observation can be made that patients with diabetes and hypertension are significantly different from all the other groups. Their *SampEn, PitEn, BinEn*, and SBP-RRI dependency level entropies (Figure 4b, Figure 8b,c, and Figure 11b) are considerably larger than in other groups, but not for SBP signal. Its composite multiscale entropy is systematically below the other groups. Its copula parameter is negative for all values of RRI delay with respect to SBP, rising a question if the blood pressure control feedback exists at all.

All these observations open new paths for further research, provided that we get the signals when the COVID outburst calms down.

## 4. Conclusions

There are still more questions than answers considering the effects of COVID-19 viral infection. The motivation for our study is a hypothesis that the complex mechanisms that alter the status of the cardiac autonomic nervous system could be tackled only by advanced multidimensional analysis of many variables, obtained both from the source cardiovascular signals, from laboratory analysis, and from detailed patient’s history. The final aim of our integral research is to extract a huge set of parameters from patients, controls, and convalescents and, applying a proper multivariable technique, to predict, and thus to prevent potential cardiovascular dysfunction that might develop long after the patients recover from COVID-19. This paper is the first step, aiming to apply entropy analysis to cardiovascular beat-to-beat signals, recorded from COVID-19 patients, and to find analytical methods that could make a distinction between the three groups of subjects—mild and severe COVID-19 patients, and healthy controls. So far, we have collected beat-to-beat signals from 116 patients with the COVID-19 disease.

We have applied sample entropy to the original signals, and to signals modified prior to *SampEn* analysis. We eliminated the amplitude distribution of SBP and RRI signals and reduced their distribution to the uniform by applying the probability integral transform. We coarsely grained the signal by averaging adjacent samples for composite multiscale entropy. We eliminated amplitudes from the signal by binary differential coding. We can differentiate patients from controls, but differentiation between mild and severe groups of patients was possible only by cross-entropy of probability transformed signals, and, surprisingly, by cross-binarized entropy. Joint symbolic dynamics entropy, with its stability expressed in a very low standard deviation, is also a candidate for COVID-19 analysis. For For the continuation of our research continued research, we have singled out *SampEn* applied to PIT signals, JSD entropy, *BinEn,* and copula parameter.

However, the true nature of COVID-19 is still unknown, and it cannot be said whether the dysfunctions of cardiovascular ANS and the changes that entropy analyses have spotted are indeed caused by a virus or just collateral damage caused by viral activity in remote parts of the body.

Further research will have to wait for the COVID outburst to calm down, as the hospitals are locked down, devoted to patients, and with no resources to spare for research. For the extended results, it is essential to get the data from convalescents, which is impossible when the acquisition equipment is in hospitals, unavailable. We shall continue with the existing signals, with non-parametric methods based on Shannon entropy–mutual information and transfer entropy, and other information-theoretic methods such as Lempel-Ziv procedures, once very popular for data compression, and nowadays used to estimate the data complexity [33,34].

## Figures and Tables

**Table 1:** List of acronyms.

Acronym	Definition
ANS	Autonomic Nervous System
ApEn	Approximate Entropy
BinEn	Binarized Entropy
BP	Blood Pressure
bpm	beats per minute
CMSE	Composite MultiScale Entropy
COVID-19	Corona Virus Disease 2019
Cross-SampEn, XSampEn	Cross-Sample Entropy
ECG	ElectroCardioGram
HR	Heart Rate
HRV	Heart Rate Variability
JSD	Joint Symbolic Dynamics
JSDSh	Shannon Joint Symbolic Dynamics Entropy
KS entropy	Kolmogorov-Sinai entropy
MSE	MultiScale Entropy
PCR	Polymerase Chain Reaction
PDF	Probability Density Function
PI-transform, PIT	Probability Integral Transform
RR	R to R peak
RRI	R to R peak Interval
SampEn	Sample Entropy
SARS-CoV-2	Severe Acute Respiratory Syndrome–Corona Virus 2
SBP	Systolic Blood Pressure
SE	Standard Error

**Table 2:** Basic parameters.

Diseases	HR	[bpm]	SBP	[mmHg]
Controls	73.27	±9.22	113.31	±11.5
Mild	81.23	±12.78	105.32	±16.57
Severe	83.24	±17.62	116.61	±18.75
Diabetes mellitus	78.22	±15.03	108.91	±12.87
Hypertension	73.04	±9.86	107.18	±14.22
Syncope	86.02	±16.05	109.41	±11.97
Diabetes + Hypertension	89.81	±20.02	138.83	±18.7
Hypertension + Syncope	76.21	±5.65	103.01	±16.14

Results are presented as mean ± standard deviation.

## Data Availability

Restrictions apply to the availability of data. Data were obtained from University Clinical Centre of the Republic of Srpska, Banja Luka (COVID-19 patients), and from University Hospital Center Bežanijska Kosa, Belgrade (healthy controls). Data are available from prof. Vlado Đajić and prof. Branislav Milovanović, with the permission of University Clinical Centre of the Republic of Srpska and University Hospital Center Bežanijska Kosa, respectively.

## Supplementary Materials

The following are available online at https://www.mdpi.com/1099-4300/23/1/87/s1, Figures S1-S184: RRI and SBP of subject #1-#184.
