# Supplementary material for: Entropy Analysis of COVID-19 Cardiovascular Signals
Source: Entropy (Basel). 2021 Jan 9;23(1):87. doi: 10.3390/e23010087 (PMC7826611; doi:10.3390/e23010087)
Supplement: Supplementary file 1 [file entropy-23-00087-s001.pdf]

COVID-19 SIGNALS

First panel: RRI time series

Second panel: SBP time series

The axis lengths for all signals are equal.

If COP and JSDSh parameters are missing, then SBP signal is not eligible for processing (too many interrupts).

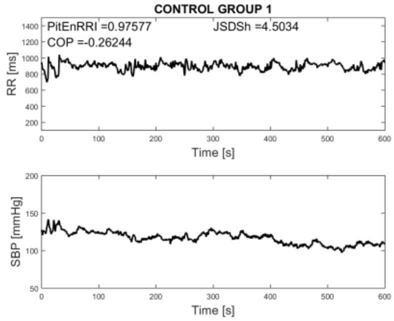
CONTROL GROUP 1
PitEnRRI =0.97577
JSDSh =4.5034
COP =-0.26244
RR [ms]
Time [s]
SBP [mmHg]
Time [s]

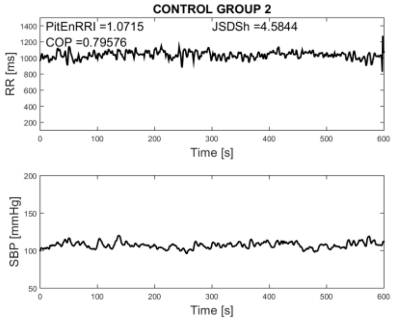
CONTROL GROUP 2
PitEnRRI =1.0715
JSDSh =4.5844
COP =0.79576
RR [ms]
Time [s]
SBP [mmHg]
Time [s]

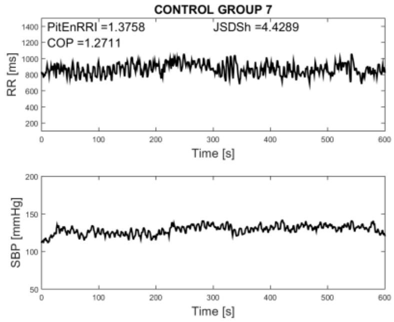
CONTROL GROUP 7
PitEnRRI =1.3758
JSDSh =4.4289
COP =1.2711
RR [ms]
Time [s]
SBP [mmHg]
Time [s]

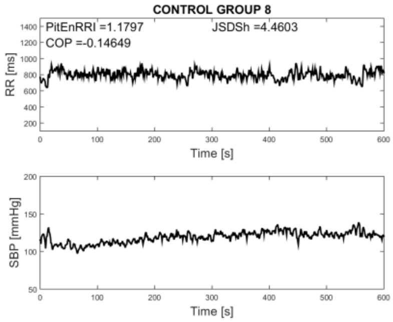
CONTROL GROUP 8
PitEnRRI =1.1797
JSDSh =4.4603
COP =-0.14649
RR [ms]
Time [s]
SBP [mmHg]
Time [s]

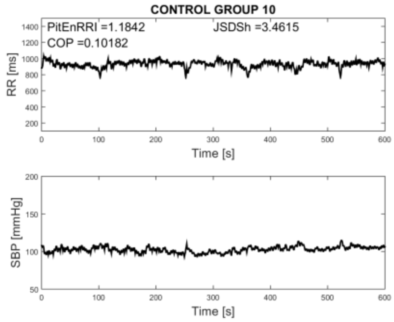
CONTROL GROUP 10
PitEnRRI =1.1842
JSDSh =3.4615
COP =0.10182
RR [ms]
Time [s]
SBP [mmHg]
Time [s]

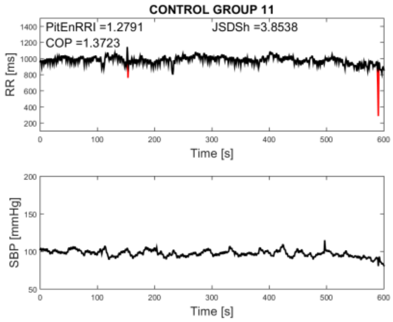
CONTROL GROUP 11
PitEnRRI =1.2791
JSDSh =3.8538
COP =1.3723
RR [ms]
Time [s]
SBP [mmHg]
Time [s]

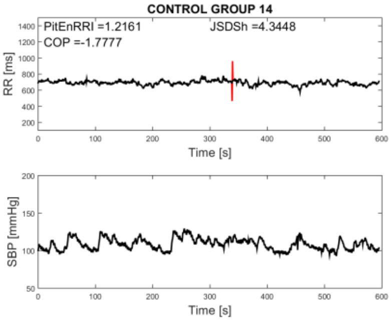
CONTROL GROUP 14
PitEnRRI =1.2161
COP =-1.7777
JSDSh =4.3448
RR [ms]
Time [s]
SBP [mmHg]
Time [s]

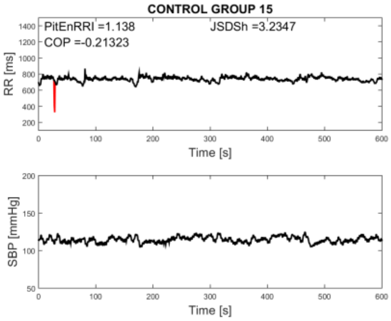
CONTROL GROUP 15
PitEnRRI =1.138
COP =-0.21323
JSDSh =3.2347
RR [ms]
Time [s]
SBP [mmHg]
Time [s]

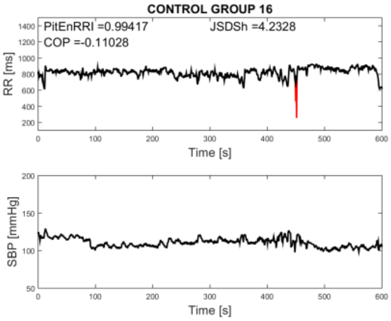
CONTROL GROUP 16
PitEnRRI =0.99417
COP =-0.11028
JSDSh =4.2328
RR [ms]
Time [s]
SBP [mmHg]
Time [s]

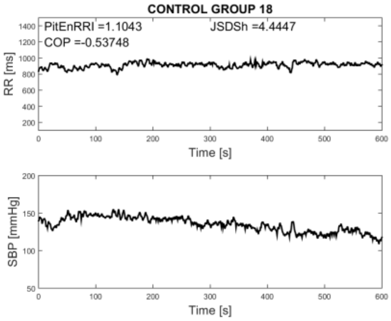
CONTROL GROUP 18
PitEnRRI =1.1043
COP =-0.53748
JSDSh =4.4447
RR [ms]
Time [s]
SBP [mmHg]
Time [s]

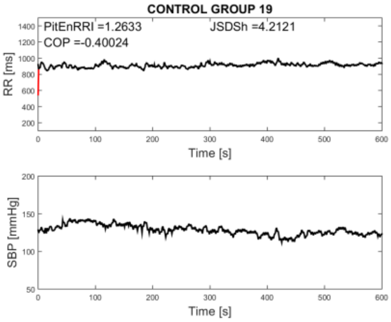
CONTROL GROUP 19
PitEnRRI =1.2633
COP =-0.40024
JSDSh =4.2121
RR [ms]
Time [s]
SBP [mmHg]
Time [s]

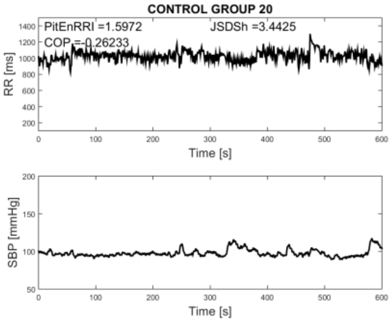
CONTROL GROUP 20
PitEnRRI =1.5972
COP =-0.26233
JSDSh =3.4425
RR [ms]
Time [s]
SBP [mmHg]
Time [s]

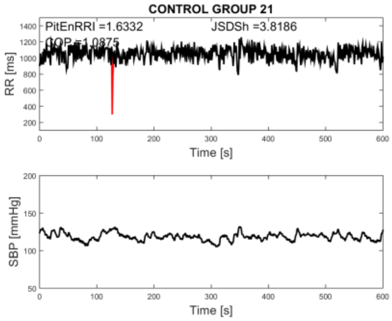
CONTROL GROUP 21
PitEnRRI =1.6332
JSDSh =3.8186
COP =1.0875
RR [ms]
Time [s]
SBP [mmHg]
Time [s]

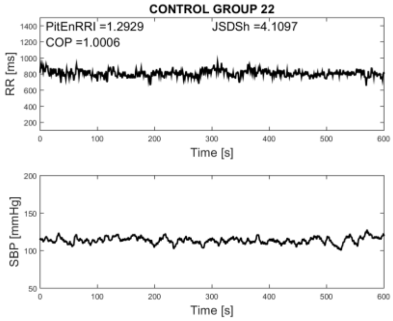
CONTROL GROUP 22
PitEnRRI =1.2929
JSDSh =4.1097
COP =1.0006
RR [ms]
Time [s]
SBP [mmHg]
Time [s]

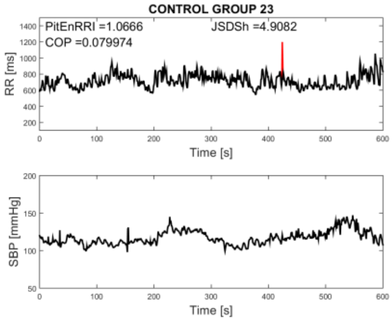
CONTROL GROUP 23
PitEnRRI =1.0666
JSDSh =4.9082
COP =0.079974
RR [ms]
Time [s]
SBP [mmHg]
Time [s]

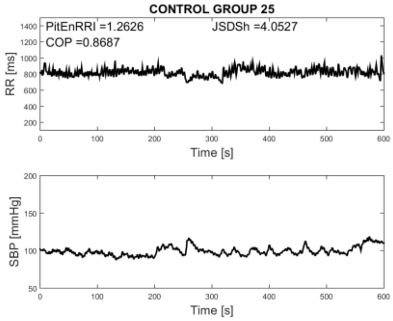
CONTROL GROUP 25
PitEnRRI =1.2626
JSDSh =4.0527
COP =0.8687
RR [ms]
Time [s]
SBP [mmHg]
Time [s]

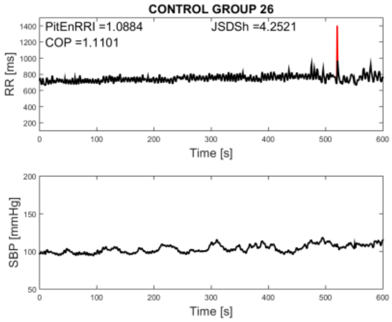
CONTROL GROUP 26
PitEnRRI =1.0884
JSDSh =4.2521
COP =1.1101
RR [ms]
Time [s]
SBP [mmHg]
Time [s]

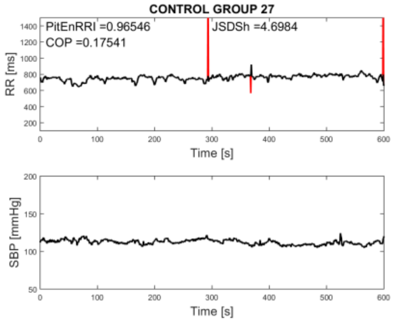
CONTROL GROUP 27
PitEnRRI =0.96546
JSDSh =4.6984
COP =0.17541
RR [ms]
Time [s]
SBP [mmHg]
Time [s]

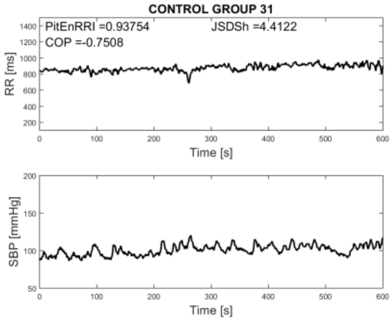
CONTROL GROUP 31
PitEnRRI =0.93754
JSDSh =4.4122
COP =-0.7508
RR [ms]
Time [s]
SBP [mmHg]
Time [s]

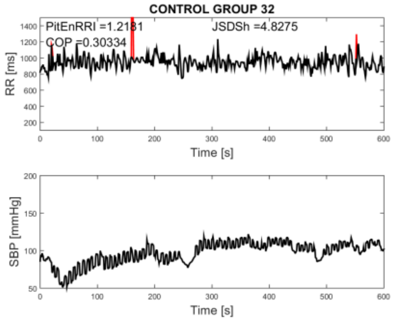
CONTROL GROUP 32
JSDSh =4.8275
RR [ms]
Time [s]
SBP [mmHg]
Time [s]

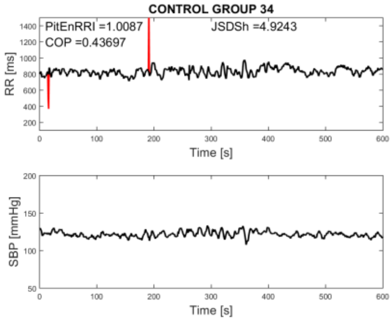
CONTROL GROUP 34
PitEnRRI =1.0087
JSDSh =4.9243
COP =0.43697
RR [ms]
Time [s]
SBP [mmHg]
Time [s]

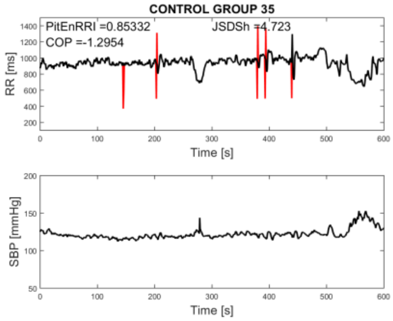
CONTROL GROUP 35
PitEnRRI =0.85332
JSDSh =4.723
COP =-1.2954
RR [ms]
Time [s]
SBP [mmHg]
Time [s]

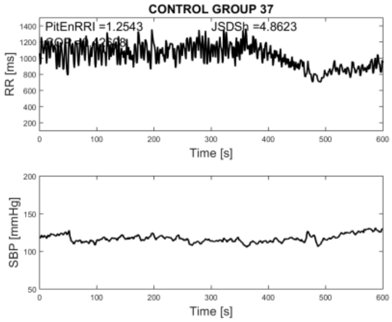
CONTROL GROUP 37
PitEnRRI =1.2543
JSDSh =4.8623
RR [ms]
Time [s]
SBP [mmHg]
Time [s]

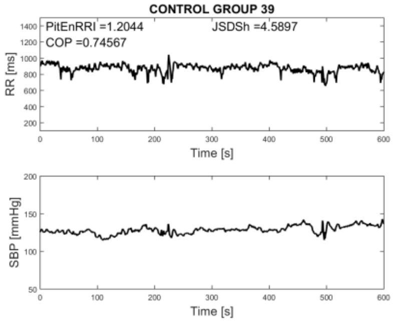
CONTROL GROUP 39
PitEnRRI =1.2044
JSDSh =4.5897
COP =0.74567
RR [ms]
Time [s]
SBP [mmHg]
Time [s]

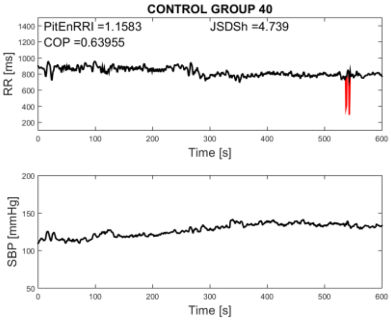
CONTROL GROUP 40
PitEnRRI =1.1583
COP =0.63955
JSDSh =4.739
RR [ms]
Time [s]
SBP [mmHg]
Time [s]

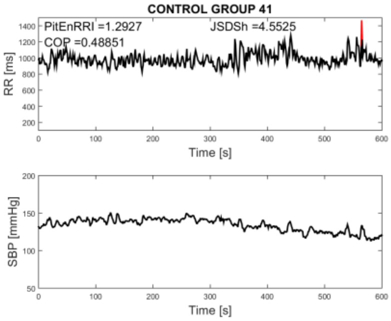
CONTROL GROUP 41
PitEnRRI =1.2927
COP =0.48851
JSDSh =4.5525
RR [ms]
Time [s]
SBP [mmHg]
Time [s]

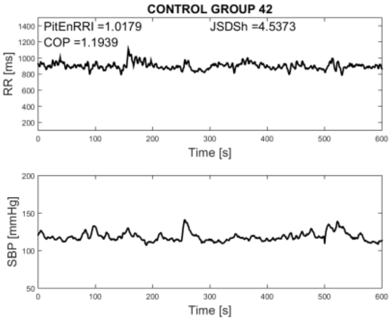
CONTROL GROUP 42
PitEnRRI =1.0179
COP =1.1939
JSDSh =4.5373
RR [ms]
Time [s]
SBP [mmHg]
Time [s]

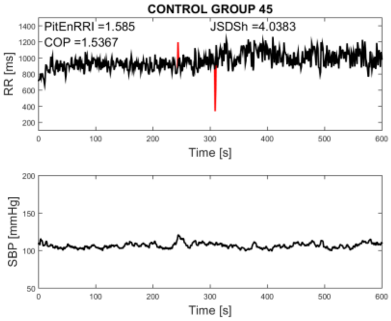
CONTROL GROUP 45
PitEnRRI =1.585
COP =1.5367
JSDSh =4.0383
RR [ms]
Time [s]
SBP [mmHg]
Time [s]

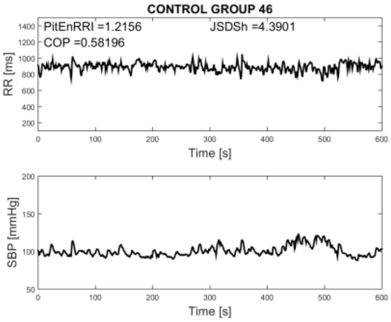
CONTROL GROUP 46
PitEnRRI =1.2156
COP =0.58196
JSDSh =4.3901
RR [ms]
Time [s]
SBP [mmHg]
Time [s]

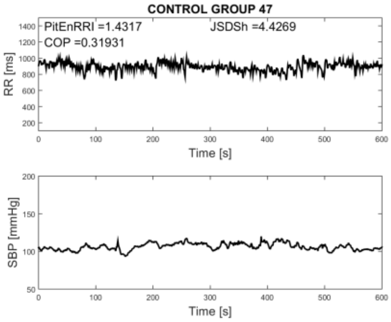
CONTROL GROUP 47
PitEnRRI =1.4317
COP =0.31931
JSDSh =4.4269
RR [ms]
Time [s]
SBP [mmHg]
Time [s]

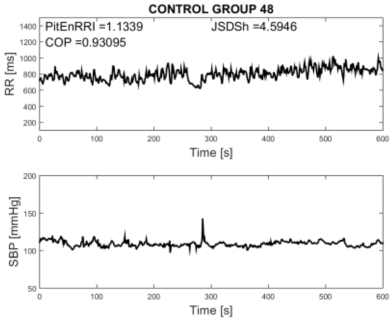
CONTROL GROUP 48
PitEnRRI =1.1339
JSDSh =4.5946
COP =0.93095
RR [ms]
Time [s]
SBP [mmHg]
Time [s]

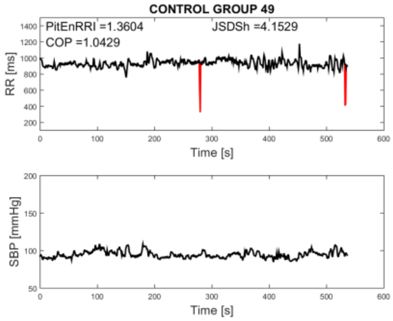
CONTROL GROUP 49
PitEnRRI =1.3604
JSDSh =4.1529
COP =1.0429
RR [ms]
Time [s]
SBP [mmHg]
Time [s]

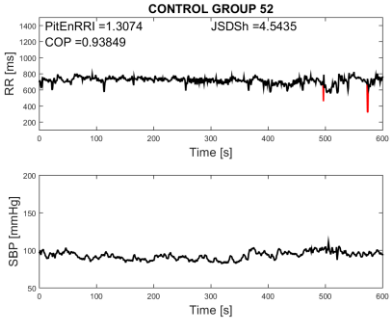
CONTROL GROUP 52
PitEnRRI =1.3074
JSDSh =4.5435
COP =0.93849
RR [ms]
Time [s]
SBP [mmHg]
Time [s]

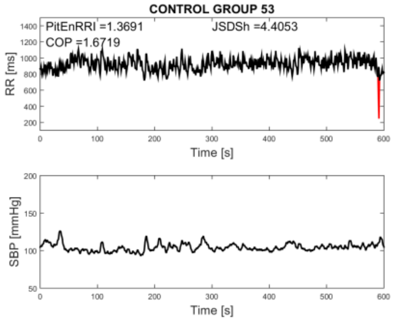
CONTROL GROUP 53
PitEnRRI =1.3691
JSDSh =4.4053
COP =1.6719
RR [ms]
Time [s]
SBP [mmHg]
Time [s]

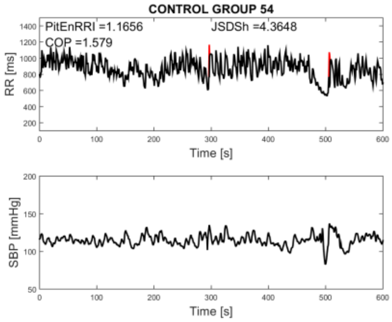
CONTROL GROUP 54
PitEnRRI =1.1656
JSDSh =4.3648
COP =1.579
RR [ms]
Time [s]
SBP [mmHg]
Time [s]

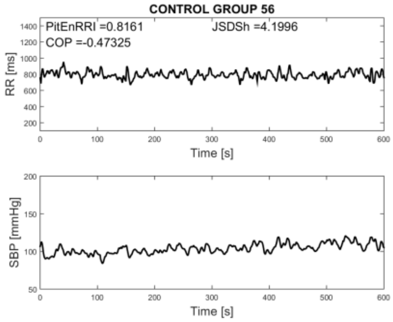
CONTROL GROUP 56
PitEnRRI =0.8161
JSDSh =4.1996
COP =-0.47325
RR [ms]
Time [s]
SBP [mmHg]
Time [s]

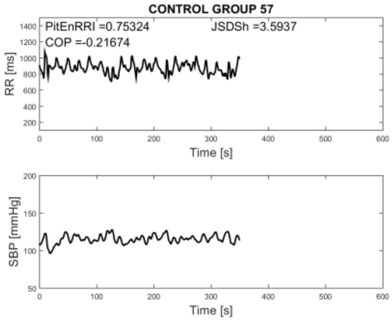
CONTROL GROUP 57
PitEnRRI =0.75324
JSDSh =3.5937
COP =-0.21674
RR [ms]
Time [s]
SBP [mmHg]
Time [s]

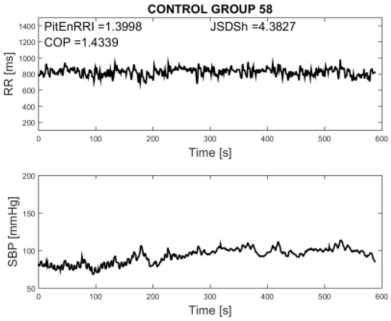
CONTROL GROUP 58
PitEnRRI =1.3998
JSDSh =4.3827
COP =1.4339
RR [ms]
Time [s]
SBP [mmHg]
Time [s]

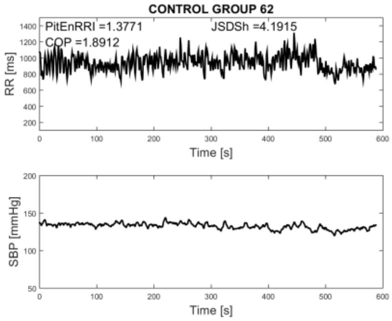
CONTROL GROUP 62
PitEnRRI =1.3771
JSDSh =4.1915
COP =1.8912
RR [ms]
Time [s]
SBP [mmHg]
Time [s]

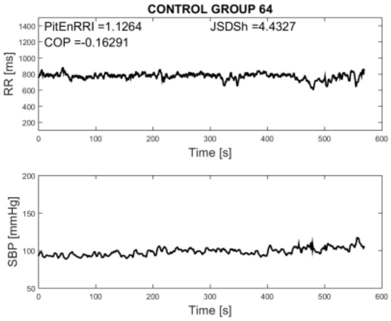
CONTROL GROUP 64
PitEnRRI =1.1264
JSDSh =4.4327
COP =-0.16291
RR [ms]
Time [s]
SBP [mmHg]
Time [s]

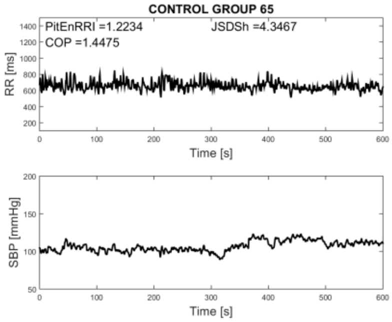
CONTROL GROUP 65
PitEnRRI =1.2234
JSDSh =4.3467
COP =1.4475
RR [ms]
Time [s]
SBP [mmHg]
Time [s]

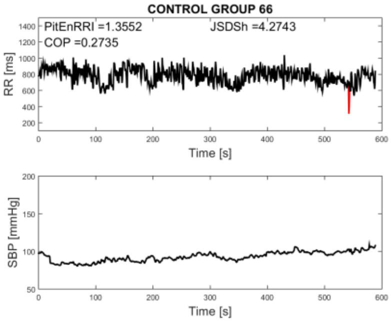
CONTROL GROUP 66
PitEnRRI =1.3552
JSDSh =4.2743
COP =0.2735
RR [ms]
Time [s]
SBP [mmHg]
Time [s]

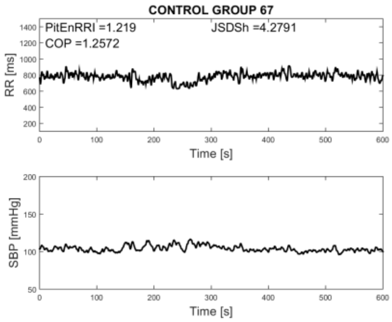
CONTROL GROUP 67
PitEnRRI =1.219
JSDSh =4.2791
COP =1.2572
RR [ms]
Time [s]
SBP [mmHg]
Time [s]

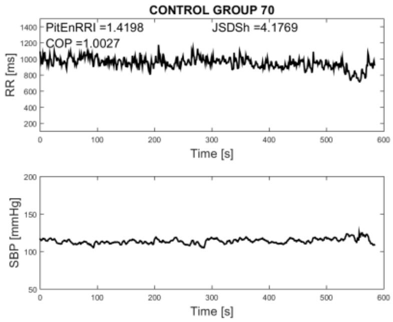
CONTROL GROUP 70
PitEnRRI =1.4198
JSDSh =4.1769
COP =1.0027
RR [ms]
Time [s]
SBP [mmHg]
Time [s]

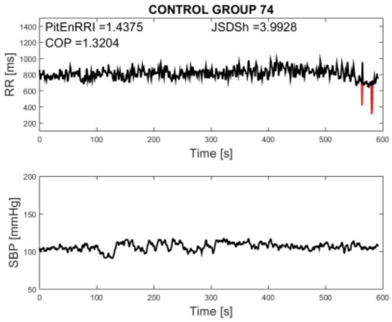
CONTROL GROUP 74
PitEnRRI =1.4375
JSDSh =3.9928
COP =1.3204
RR [ms]
Time [s]
SBP [mmHg]
Time [s]

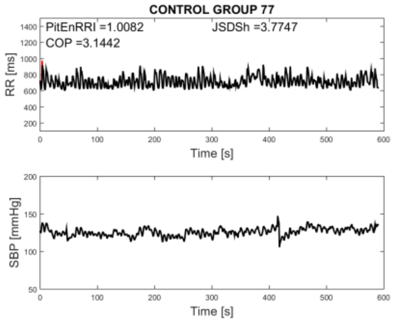
CONTROL GROUP 77
PitEnRRI =1.0082
JSDSh =3.7747
COP =3.1442
RR [ms]
Time [s]
SBP [mmHg]
Time [s]

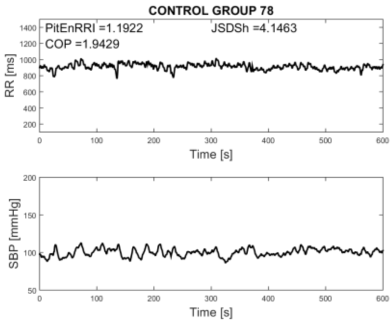
CONTROL GROUP 78
PitEnRRI =1.1922
JSDSh =4.1463
COP =1.9429
RR [ms]
Time [s]
SBP [mmHg]
Time [s]

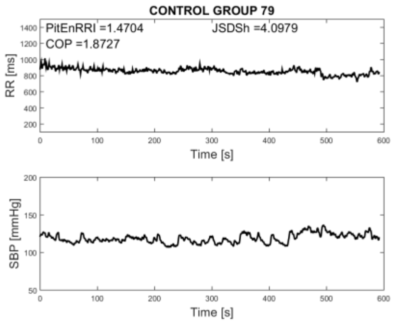
CONTROL GROUP 79
PitEnRRI =1.4704
JSDSh =4.0979
COP =1.8727
RR [ms]
Time [s]
SBP [mmHg]
Time [s]

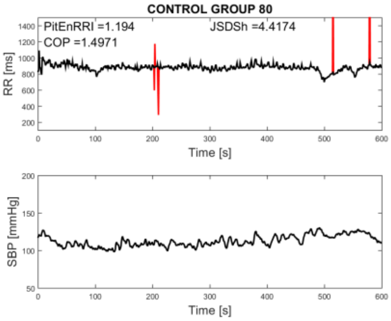
CONTROL GROUP 80
PitEnRRI =1.194
JSDSh =4.4174
COP =1.4971
RR [ms]
Time [s]
SBP [mmHg]
Time [s]

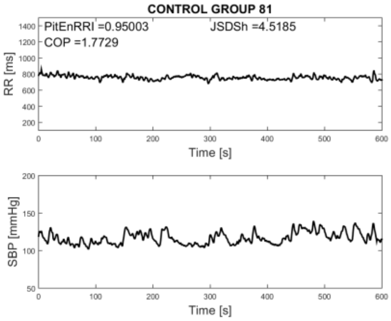
CONTROL GROUP 81
PitEnRRI =0.95003
JSDSh =4.5185
COP =1.7729
RR [ms]
Time [s]
SBP [mmHg]
Time [s]

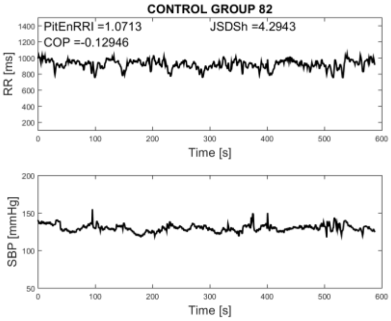
CONTROL GROUP 82
PitEnRRI =1.0713
JSDSh =4.2943
COP =-0.12946
RR [ms]
Time [s]
SBP [mmHg]
Time [s]

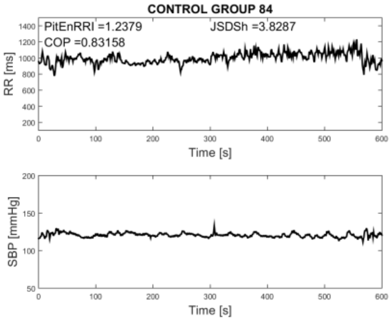
CONTROL GROUP 84
PitEnRRI =1.2379
JSDSh =3.8287
COP =0.83158
RR [ms]
Time [s]
SBP [mmHg]
Time [s]

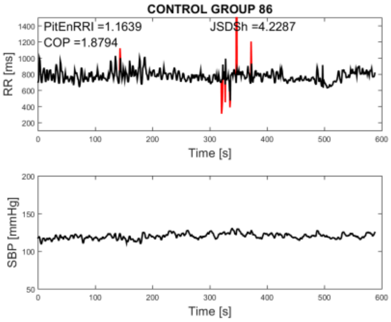
CONTROL GROUP 86
PitEnRRI =1.1639
JSDSh =4.2287
COP =1.8794
RR [ms]
Time [s]
SBP [mmHg]
Time [s]

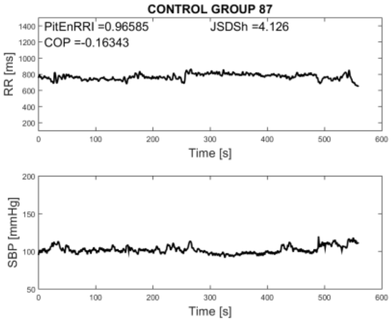
CONTROL GROUP 87
PitEnRRI =0.96585
JSDSh =4.126
COP =-0.16343
RR [ms]
Time [s]
SBP [mmHg]
Time [s]

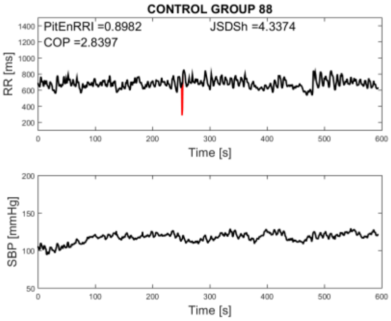
CONTROL GROUP 88
PitEnRRI =0.8982
JSDSh =4.3374
COP =2.8397
RR [ms]
Time [s]
SBP [mmHg]
Time [s]

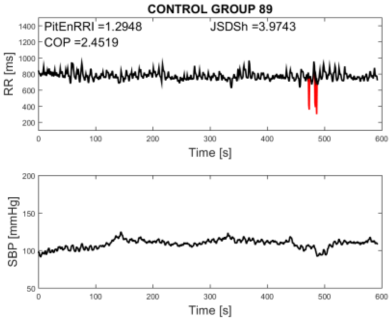
CONTROL GROUP 89
PitEnRRI =1.2948
JSDSh =3.9743
COP =2.4519
RR [ms]
Time [s]
SBP [mmHg]
Time [s]

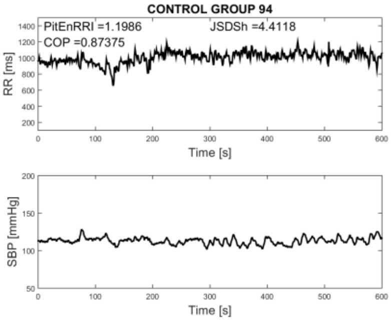
CONTROL GROUP 94
PitEnRRI =1.1986
JSDSh =4.4118
COP =0.87375
RR [ms]
Time [s]
SBP [mmHg]
Time [s]

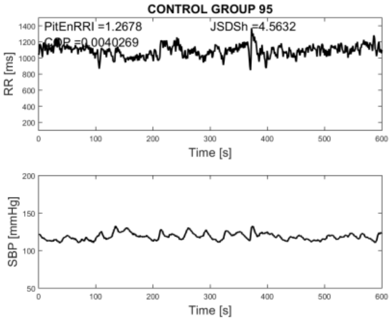
CONTROL GROUP 95
PitEnRRI =1.2678
JSDSh =4.5632
COP =0.0040269
RR [ms]
Time [s]
SBP [mmHg]
Time [s]

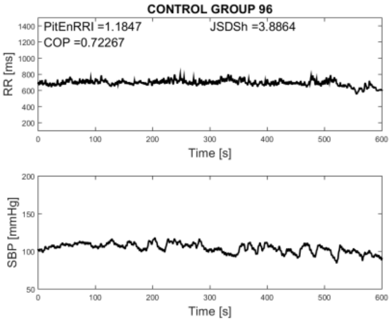
CONTROL GROUP 96
PitEnRRI =1.1847
JSDSh =3.8864
COP =0.72267
RR [ms]
Time [s]
SBP [mmHg]
Time [s]

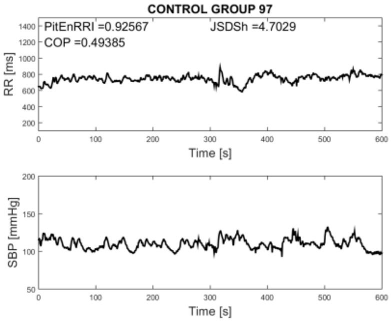
CONTROL GROUP 97
PitEnRRI =0.92567
JSDSh =4.7029
COP =0.49385
RR [ms]
Time [s]
SBP [mmHg]
Time [s]

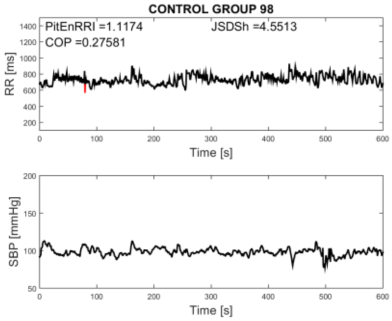
CONTROL GROUP 98
PitEnRRI =1.1174
COP =0.27581
JSDSh =4.5513
RR [ms]
Time [s]
SBP [mmHg]
Time [s]

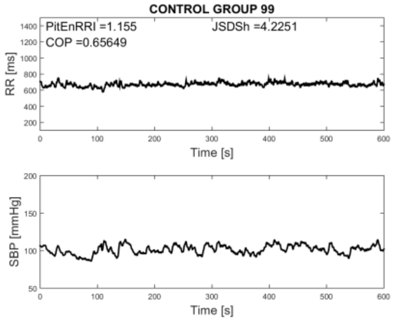
CONTROL GROUP 99
PitEnRRI =1.155
COP =0.65649
JSDSh =4.2251
RR [ms]
Time [s]
SBP [mmHg]
Time [s]

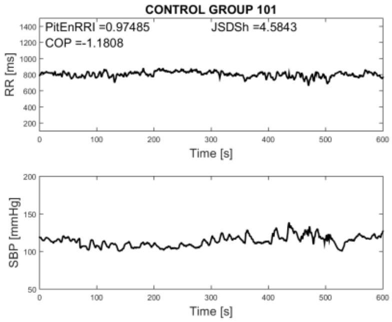
CONTROL GROUP 101
PitEnRRI =0.97485
COP =-1.1808
JSDSh =4.5843
RR [ms]
Time [s]
SBP [mmHg]
Time [s]

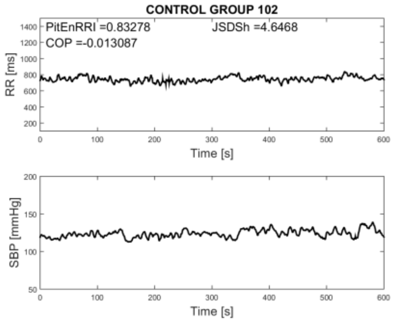
CONTROL GROUP 102
PitEnRRI =0.83278
COP =-0.013087
JSDSh =4.6468
RR [ms]
Time [s]
SBP [mmHg]
Time [s]

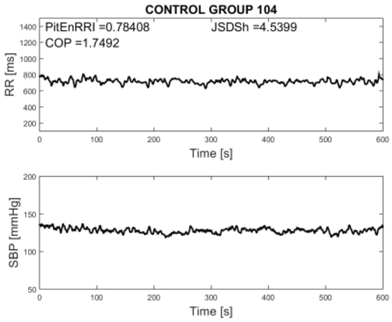
CONTROL GROUP 104
PitEnRRI =0.78408
COP =1.7492
JSDSh =4.5399
RR [ms]
Time [s]
SBP [mmHg]
Time [s]

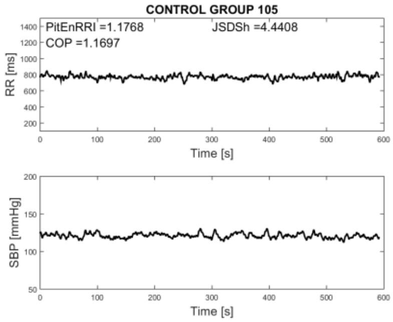
CONTROL GROUP 105
PitEnRRI =1.1768
COP =1.1697
JSDSh =4.4408
RR [ms]
Time [s]
SBP [mmHg]
Time [s]

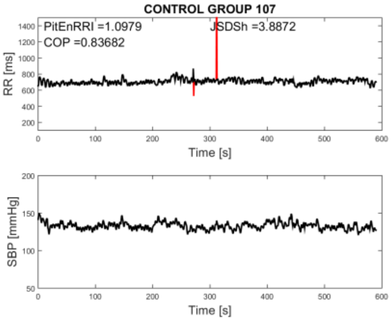
CONTROL GROUP 107
PitEnRRI =1.0979
COP =0.83682
JSDSh =3.8872
RR [ms]
Time [s]
SBP [mmHg]
Time [s]

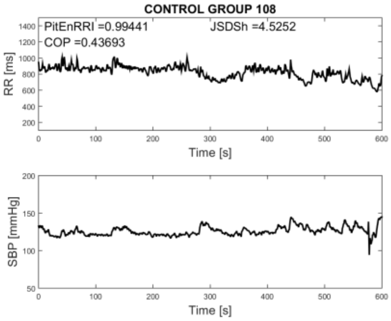
CONTROL GROUP 108
PitEnRRI =0.99441
COP =0.43693
JSDSh =4.5252
RR [ms]
Time [s]
SBP [mmHg]
Time [s]

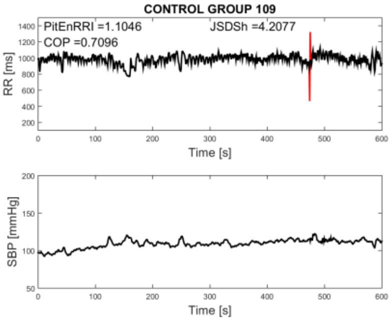
CONTROL GROUP 109
PitEnRRI =1.1046
COP =0.7096
JSDSh =4.2077
RR [ms]
Time [s]
SBP [mmHg]
Time [s]

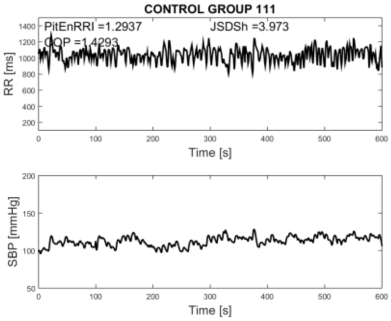
CONTROL GROUP 111
PitEnRRI =1.2937
COP =1.4293
JSDSh =3.973
RR [ms]
Time [s]
SBP [mmHg]
Time [s]

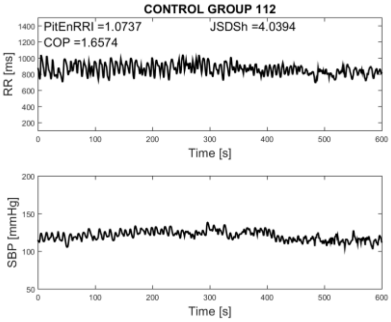
CONTROL GROUP 112
PitEnRRI =1.0737
COP =1.6574
JSDSh =4.0394
RR [ms]
Time [s]
SBP [mmHg]
Time [s]

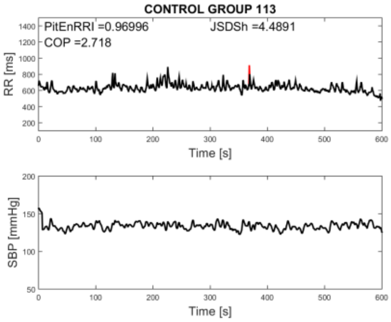
CONTROL GROUP 113
PitEnRRI =0.96996
COP =2.718
JSDSh =4.4891
RR [ms]
Time [s]
SBP [mmHg]
Time [s]

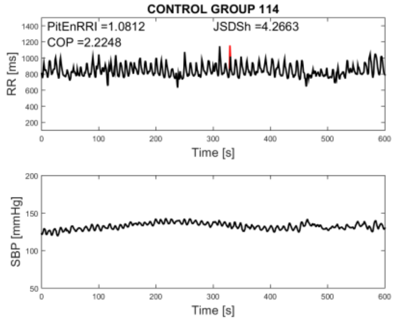
CONTROL GROUP 114
PitEnRRI =1.0812
JSDSh =4.2663
COP =2.2248
RR [ms]
Time [s]
SBP [mmHg]
Time [s]

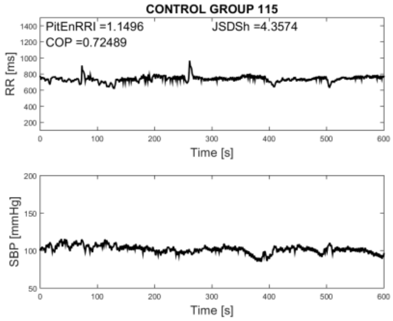
CONTROL GROUP 115
PitEnRRI =1.1496
JSDSh =4.3574
COP =0.72489
RR [ms]
Time [s]
SBP [mmHg]
Time [s]

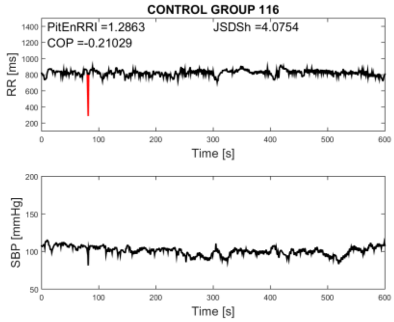
CONTROL GROUP 116
PitEnRRI =1.2863
JSDSh =4.0754
COP =-0.21029
RR [ms]
Time [s]
SBP [mmHg]
Time [s]

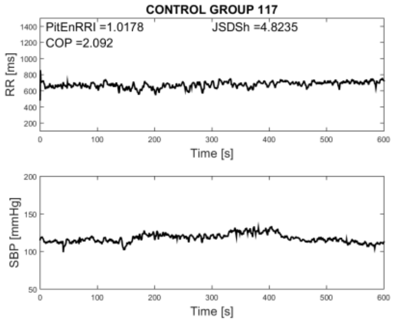
CONTROL GROUP 117
PitEnRRI =1.0178
JSDSh =4.8235
COP =2.092
RR [ms]
Time [s]
SBP [mmHg]
Time [s]

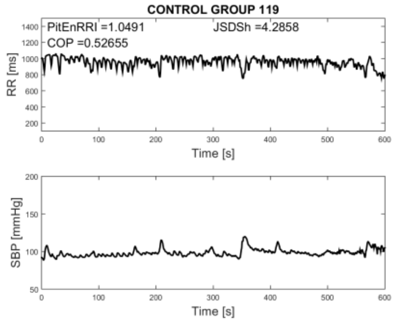
CONTROL GROUP 119
PitEnRRI =1.0491
JSDSh =4.2858
COP =0.52655
RR [ms]
Time [s]
SBP [mmHg]
Time [s]

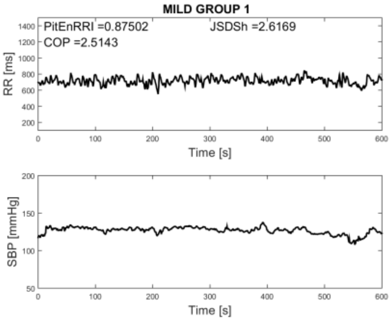
MILD GROUP 1
PitEnRRI =0.87502
JSDSh =2.6169
COP =2.5143
RR [ms]
Time [s]
SBP [mmHg]
Time [s]

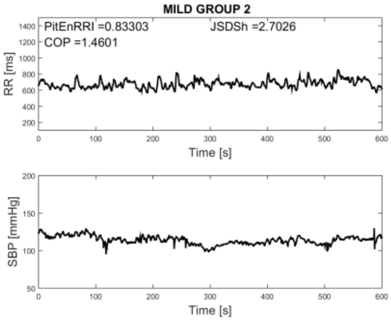
MILD GROUP 2
PitEnRRI =0.83303
JSDSh =2.7026
COP =1.4601
RR [ms]
Time [s]
SBP [mmHg]
Time [s]

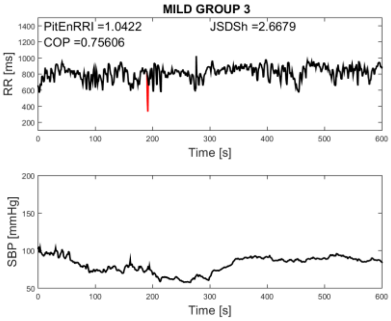
MILD GROUP 3
PitEnRRI =1.0422
JSDSh =2.6679
COP =0.75606
RR [ms]
Time [s]
SBP [mmHg]
Time [s]

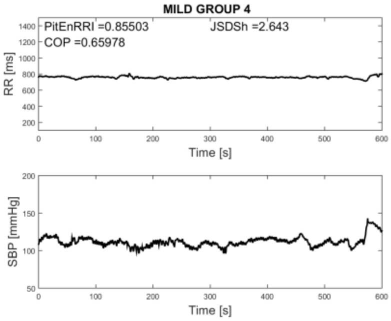
MILD GROUP 4
PitEnRRI =0.85503
JSDSh =2.643
COP =0.65978
RR [ms]
Time [s]
SBP [mmHg]
Time [s]

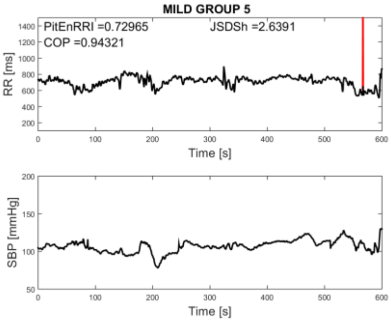
MILD GROUP 5
PitEnRRI =0.72965
JSDSh =2.6391
COP =0.94321
RR [ms]
Time [s]
SBP [mmHg]
Time [s]

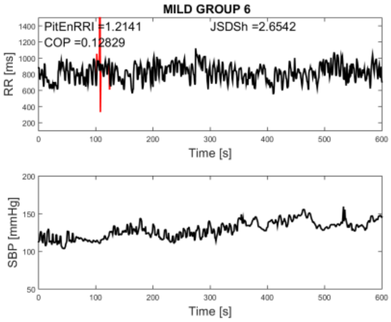
MILD GROUP 6
PitEnRRI =1.2141
JSDSh =2.6542
COP =0.12829
RR [ms]
Time [s]
SBP [mmHg]
Time [s]

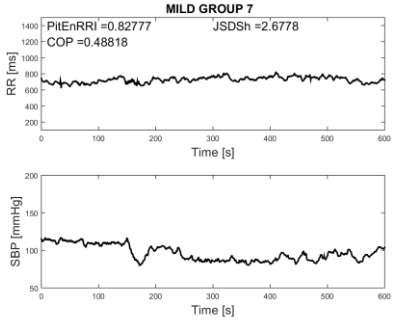
MILD GROUP 7
PitEnRRI =0.82777
JSDSh =2.6778
COP =0.48818
RR [ms]
Time [s]
SBP [mmHg]
Time [s]

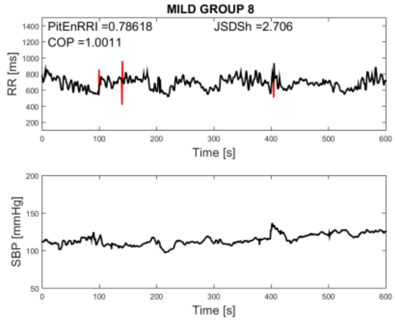
MILD GROUP 8
PitEnRRI =0.78618
JSDSh =2.706
COP =1.0011
RR [ms]
Time [s]
SBP [mmHg]
Time [s]

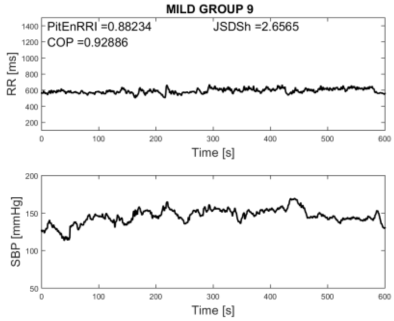
MILD GROUP 9
PitEnRRI =0.88234
JSDSh =2.6565
COP =0.92886
RR [ms]
Time [s]
SBP [mmHg]
Time [s]

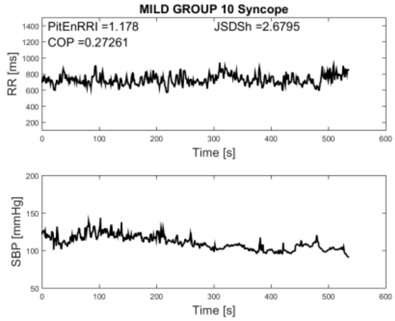
MILD GROUP 10 Syncope
PitEnRRI =1.178
JSDSh =2.6795
COP =0.27261
RR [ms]
Time [s]
SBP [mmHg]
Time [s]

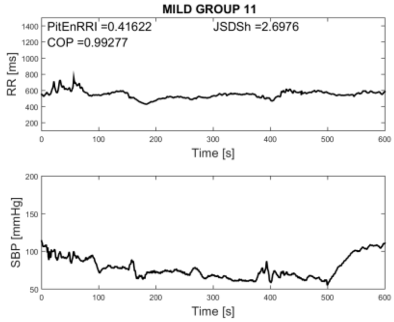
MILD GROUP 11
PitEnRRI =0.41622
JSDSh =2.6976
COP =0.99277
RR [ms]
Time [s]
SBP [mmHg]
Time [s]

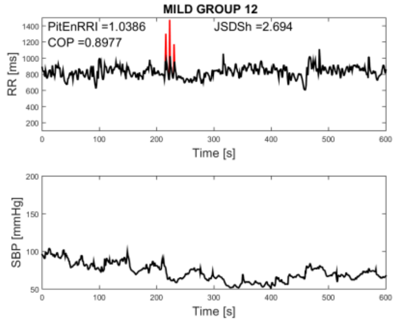
MILD GROUP 12
PitEnRRI =1.0386
JSDSh =2.694
COP =0.8977
RR [ms]
Time [s]
SBP [mmHg]
Time [s]

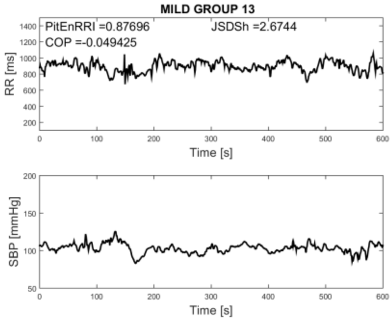
MILD GROUP 13
PitEnRRI =0.87696
JSDSh =2.6744
COP =-0.049425
RR [ms]
Time [s]
SBP [mmHg]
Time [s]

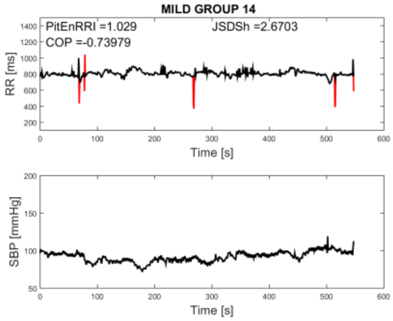
MILD GROUP 14
PitEnRRI =1.029
JSDSh =2.6703
COP =-0.73979
RR [ms]
Time [s]
SBP [mmHg]
Time [s]

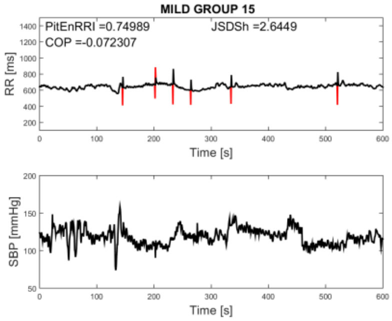
MILD GROUP 15
PitEnRRI =0.74989
JSDSh =2.6449
COP =-0.072307
RR [ms]
Time [s]
SBP [mmHg]
Time [s]

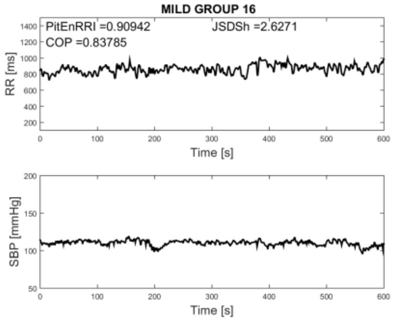
MILD GROUP 16
PitEnRRI =0.90942
JSDSh =2.6271
COP =0.83785
RR [ms]
Time [s]
SBP [mmHg]
Time [s]

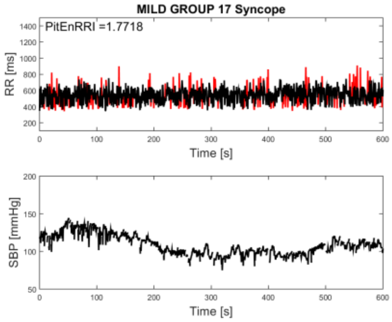
MILD GROUP 17 Syncope
PitEnRRI =1.7718
RR [ms]
Time [s]
SBP [mmHg]
Time [s]

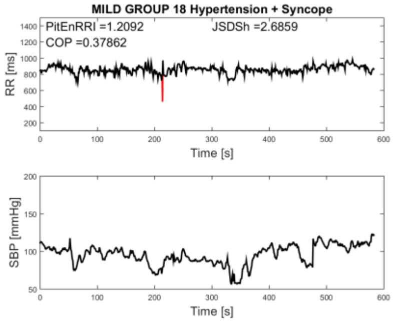
MILD GROUP 18 Hypertension + Syncope
PitEnRRI =1.2092
JSDSh =2.6859
COP =0.37862
RR [ms]
Time [s]
SBP [mmHg]
Time [s]

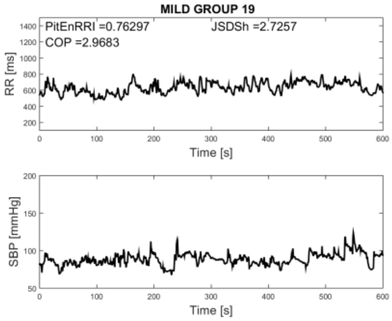
MILD GROUP 19
PitEnRRI =0.76297
JSDSh =2.7257
COP =2.9683
RR [ms]
Time [s]
SBP [mmHg]
Time [s]

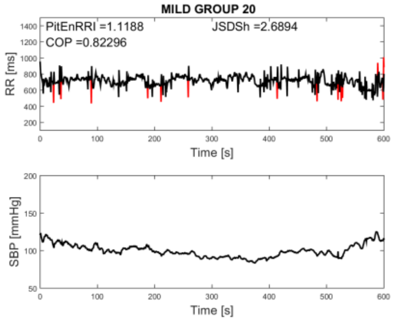
MILD GROUP 20
PitEnRRI =1.1188
JSDSh =2.6894
COP =0.82296
RR [ms]
Time [s]
SBP [mmHg]
Time [s]

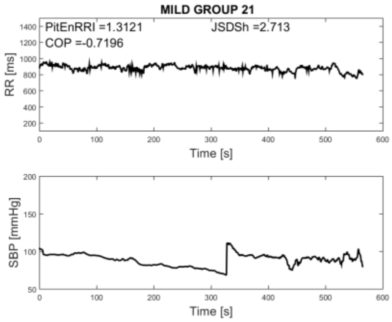
MILD GROUP 21
PitEnRRI =1.3121
JSDSh =2.713
COP =-0.7196
RR [ms]
Time [s]
SBP [mmHg]
Time [s]

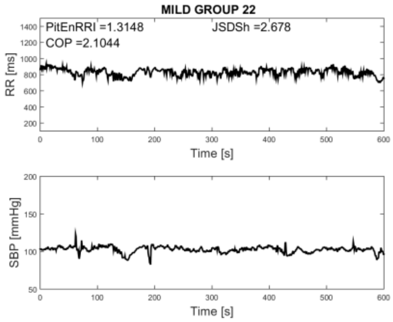
MILD GROUP 22
PitEnRRI =1.3148
JSDSh =2.678
COP =2.1044
RR [ms]
Time [s]
SBP [mmHg]
Time [s]

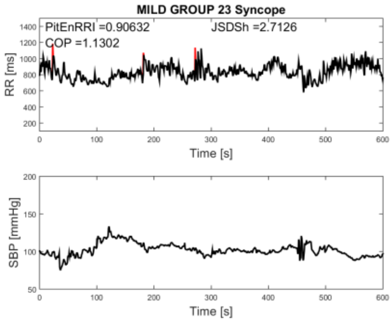
MILD GROUP 23 Syncope
PitEnRRI =0.90632
JSDSh =2.7126
COP =1.1302
RR [ms]
Time [s]
SBP [mmHg]
Time [s]

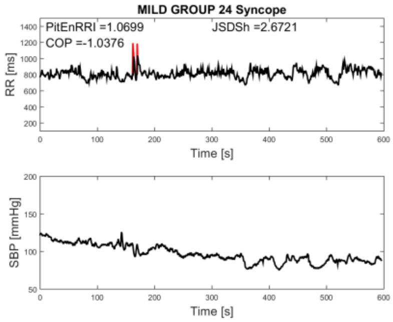
MILD GROUP 24 Syncope
PitEnRRI =1.0699
JSDSh =2.6721
COP =-1.0376
RR [ms]
Time [s]
SBP [mmHg]
Time [s]

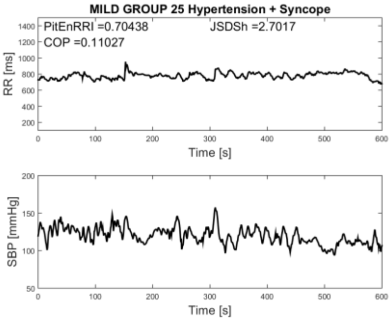
MILD GROUP 25 Hypertension + Syncope
PitEnRRI =0.70438
JSDSh =2.7017
COP =0.11027
RR [ms]
Time [s]
SBP [mmHg]
Time [s]

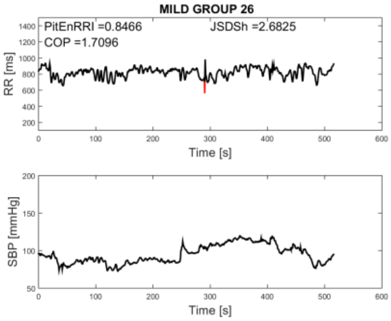
MILD GROUP 26
PitEnRRI =0.8466
JSDSh =2.6825
COP =1.7096
RR [ms]
Time [s]
SBP [mmHg]
Time [s]

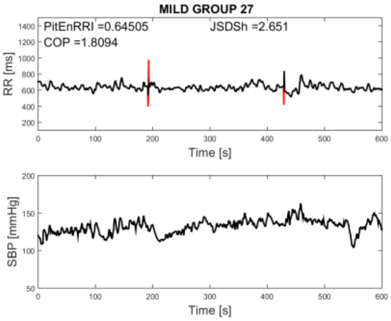
MILD GROUP 27
PitEnRRI =0.64505
JSDSh =2.651
COP =1.8094
RR [ms]
Time [s]
SBP [mmHg]
Time [s]

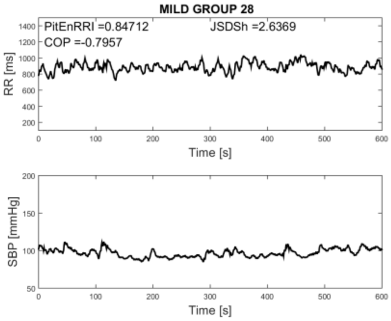
MILD GROUP 28
PitEnRRI =0.84712
JSDSh =2.6369
COP =-0.7957
RR [ms]
Time [s]
SBP [mmHg]
Time [s]

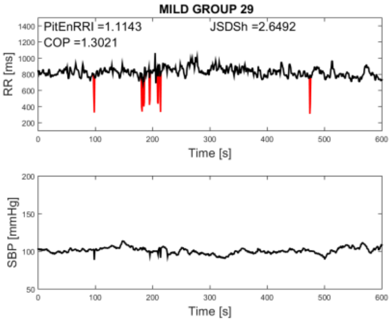
MILD GROUP 29
PitEnRRI =1.1143
JSDSh =2.6492
COP =1.3021
RR [ms]
Time [s]
SBP [mmHg]
Time [s]

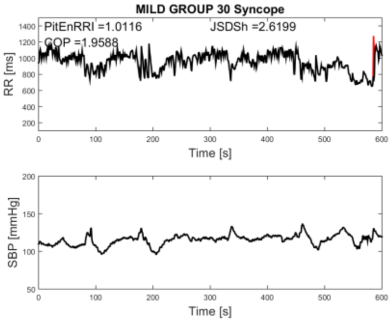
MILD GROUP 30 Syncope
PitEnRRI =1.0116
JSDSh =2.6199
COP =1.9588
RR [ms]
Time [s]
SBP [mmHg]
Time [s]

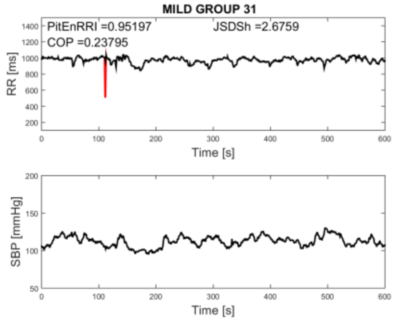
MILD GROUP 31
PitEnRRI =0.95197
JSDSh =2.6759
COP =0.23795
RR [ms]
Time [s]
SBP [mmHg]
Time [s]

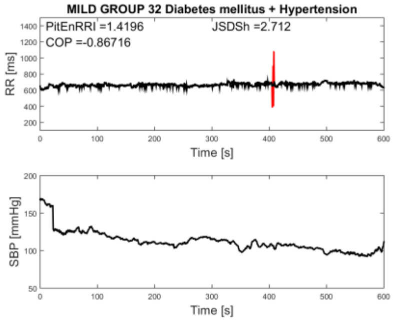
MILD GROUP 32 Diabetes mellitus + Hypertension
PitEnRRI =1.4196
JSDSh =2.712
COP =-0.86716
RR [ms]
Time [s]
SBP [mmHg]
Time [s]

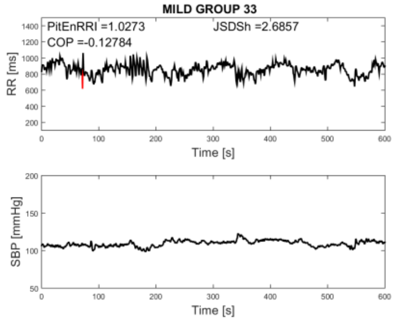
MILD GROUP 33
PitEnRRI =1.0273
JSDSh =2.6857
COP =-0.12784
RR [ms]
Time [s]
SBP [mmHg]
Time [s]

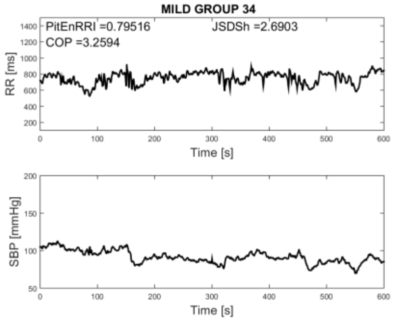
MILD GROUP 34
PitEnRRI =0.79516
JSDSh =2.6903
COP =3.2594
RR [ms]
Time [s]
SBP [mmHg]
Time [s]

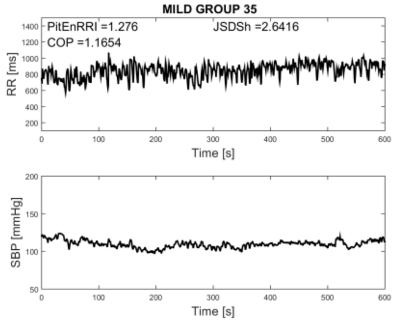
MILD GROUP 35
PitEnRRI =1.276
JSDSh =2.6416
COP =1.1654
RR [ms]
Time [s]
SBP [mmHg]
Time [s]

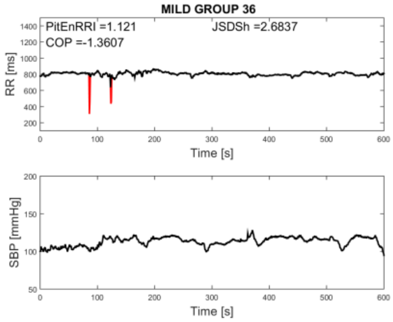
MILD GROUP 36
PitEnRRI =1.121
JSDSh =2.6837
COP =-1.3607
RR [ms]
Time [s]
SBP [mmHg]
Time [s]

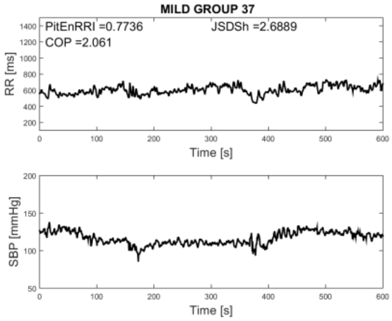
MILD GROUP 37
PitEnRRI =0.7736
JSDSh =2.6889
COP =2.061
RR [ms]
Time [s]
SBP [mmHg]
Time [s]

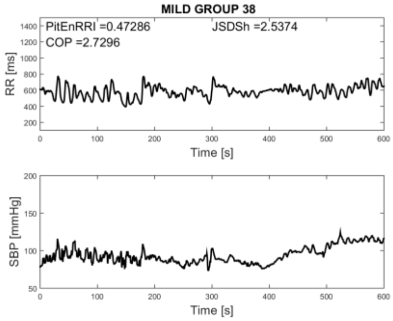
MILD GROUP 38
PitEnRRI =0.47286
JSDSh =2.5374
COP =2.7296
RR [ms]
Time [s]
SBP [mmHg]
Time [s]

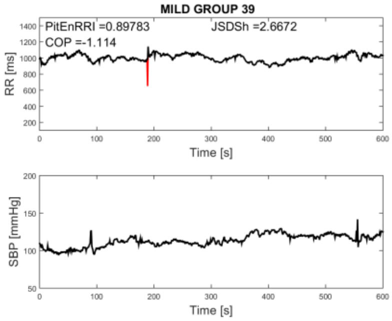
MILD GROUP 39
PitEnRRI =0.89783
JSDSh =2.6672
COP =-1.114
RR [ms]
Time [s]
SBP [mmHg]
Time [s]

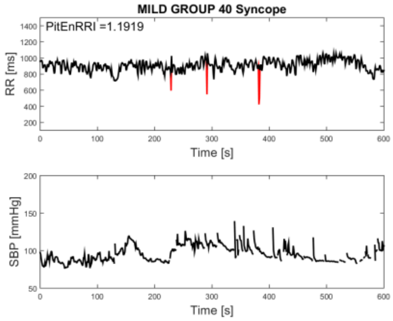
MILD GROUP 40 Syncope
PitEnRRI =1.1919
RR [ms]
Time [s]
SBP [mmHg]
Time [s]

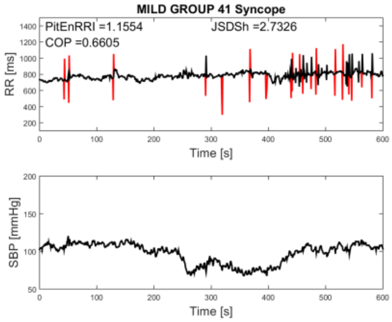
MILD GROUP 41 Syncope
PitEnRRI =1.1554
JSDSh =2.7326
COP =0.6605
RR [ms]
Time [s]
SBP [mmHg]
Time [s]

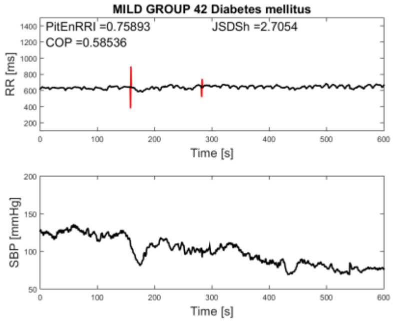
MILD GROUP 42 Diabetes mellitus
PitEnRRI =0.75893
JSDSh =2.7054
COP =0.58536
RR [ms]
Time [s]
SBP [mmHg]
Time [s]

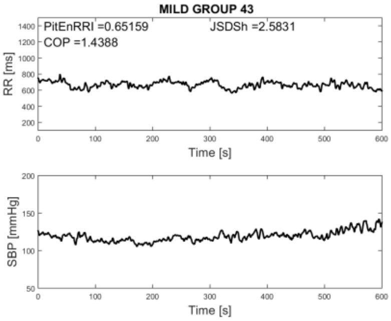
MILD GROUP 43
PitEnRRI =0.65159
JSDSh =2.5831
COP =1.4388
RR [ms]
Time [s]
SBP [mmHg]
Time [s]

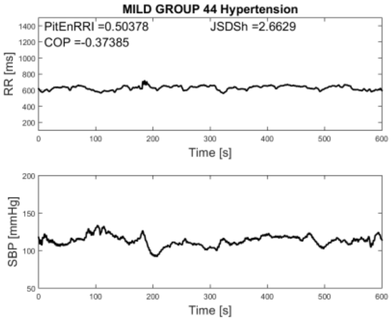
MILD GROUP 44 Hypertension
PitEnRRI =0.50378
JSDSh =2.6629
COP =-0.37385
RR [ms]
Time [s]
SBP [mmHg]
Time [s]

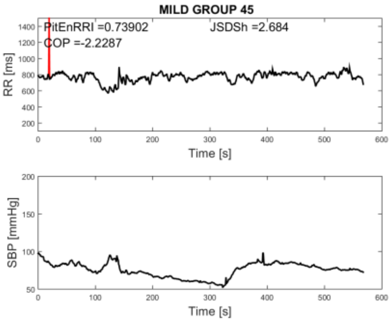
MILD GROUP 45
PitEnRRI =0.73902
JSDSh =2.684
COP =-2.2287
RR [ms]
Time [s]
SBP [mmHg]
Time [s]

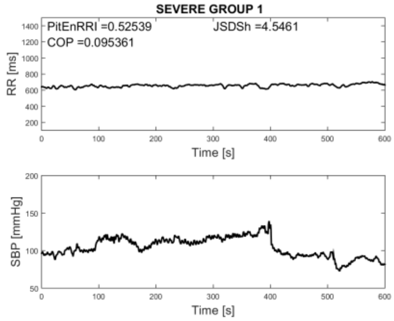
SEVERE GROUP 1
PitEnRRI =0.52539
JSDSh =4.5461
COP =0.095361
RR [ms]
Time [s]
SBP [mmHg]
Time [s]

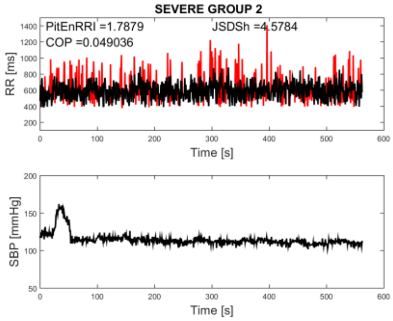
SEVERE GROUP 2
PitEnRRI =1.7879
JSDSh =4.5784
COP =0.049036
RR [ms]
Time [s]
SBP [mmHg]
Time [s]

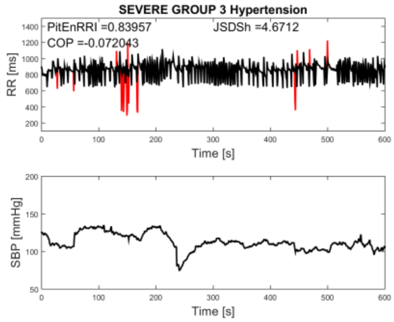
SEVERE GROUP 3 Hypertension
PitEnRRI =0.83957
JSDSh =4.6712
COP =-0.072043
RR [ms]
Time [s]
SBP [mmHg]
Time [s]

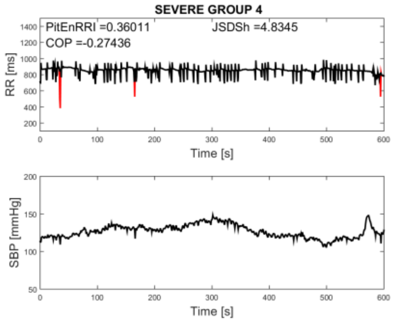
SEVERE GROUP 4
PitEnRRI =0.36011
JSDSh =4.8345
COP =-0.27436
RR [ms]
Time [s]
SBP [mmHg]
Time [s]

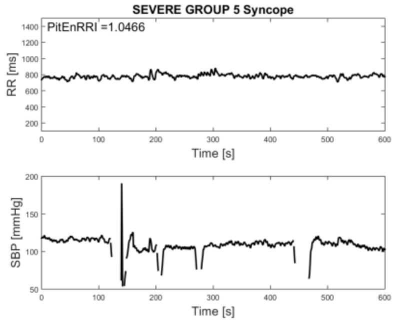
SEVERE GROUP 5 Syncope
PitEnRRI =1.0466
RR [ms]
Time [s]
SBP [mmHg]
Time [s]

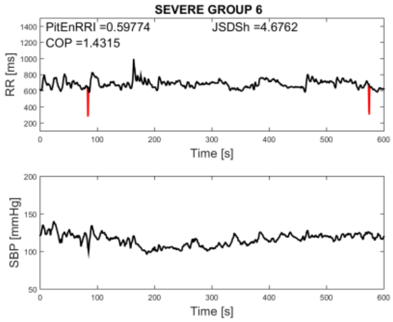
SEVERE GROUP 6
PitEnRRI =0.59774
JSDSh =4.6762
COP =1.4315
RR [ms]
Time [s]
SBP [mmHg]
Time [s]

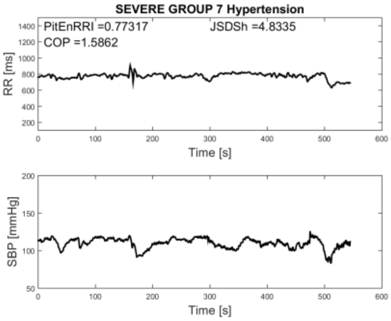
SEVERE GROUP 7 Hypertension
PitEnRRI =0.77317
JSDSh =4.8335
COP =1.5862
RR [ms]
Time [s]
SBP [mmHg]
Time [s]

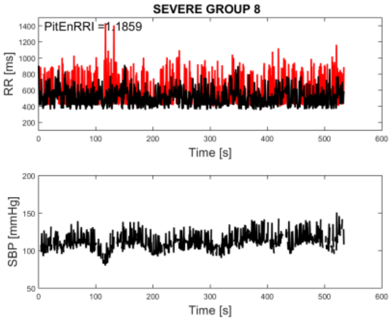
SEVERE GROUP 8
PitEnRRI =1.1859
RR [ms]
Time [s]
SBP [mmHg]
Time [s]

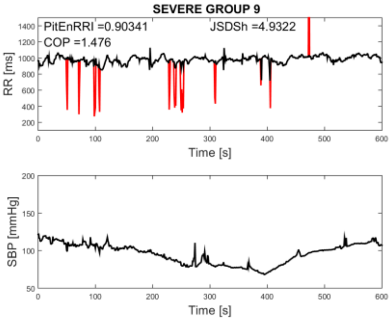
SEVERE GROUP 9
PitEnRRI =0.90341
JSDSh =4.9322
COP =1.476
RR [ms]
Time [s]
SBP [mmHg]
Time [s]

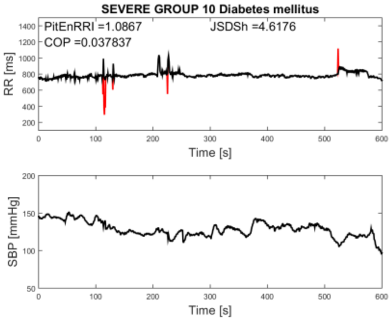
SEVERE GROUP 10 Diabetes mellitus
PitEnRRI =1.0867
JSDSh =4.6176
COP =0.037837
RR [ms]
Time [s]
SBP [mmHg]
Time [s]

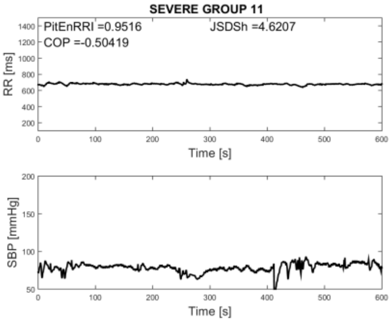
SEVERE GROUP 11
PitEnRRI =0.9516
JSDSh =4.6207
COP =-0.50419
RR [ms]
Time [s]
SBP [mmHg]
Time [s]

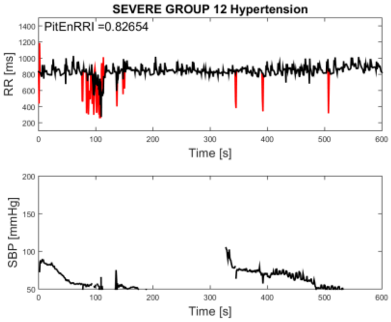
SEVERE GROUP 12 Hypertension
PitEnRRI =0.82654
RR [ms]
Time [s]
SBP [mmHg]
Time [s]

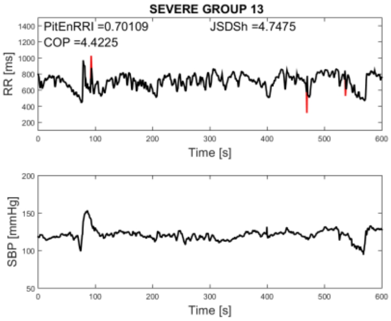
SEVERE GROUP 13
PitEnRRI =0.70109
JSDSh =4.7475
COP =4.4225
RR [ms]
Time [s]
SBP [mmHg]
Time [s]

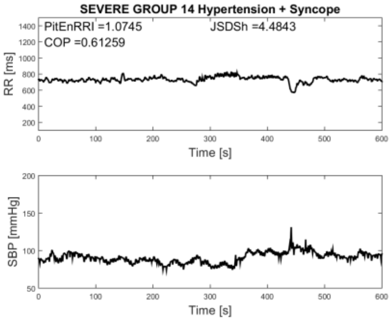
SEVERE GROUP 14 Hypertension + Syncope
PitEnRRI =1.0745
JSDSh =4.4843
COP =0.61259
RR [ms]
Time [s]
SBP [mmHg]
Time [s]

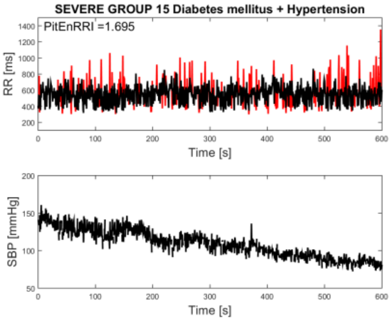
SEVERE GROUP 15 Diabetes mellitus + Hypertension
PitEnRRI =1.695
RR [ms]
Time [s]
SBP [mmHg]
Time [s]

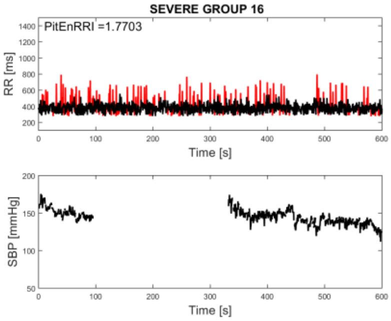
SEVERE GROUP 16
PitEnRRI =1.7703
RR [ms]
Time [s]
SBP [mmHg]
Time [s]

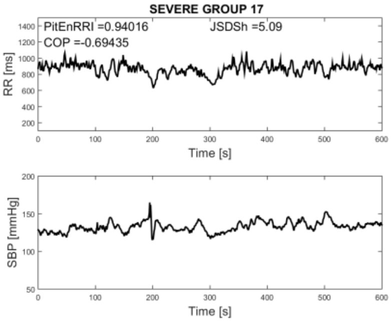
SEVERE GROUP 17
PitEnRRI =0.94016
JSDSh =5.09
COP =-0.69435
RR [ms]
Time [s]
SBP [mmHg]
Time [s]

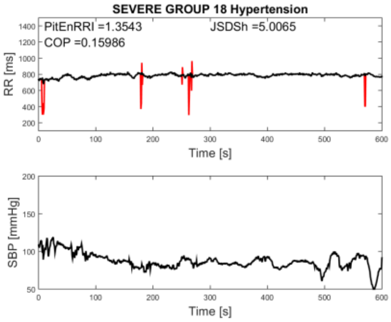
SEVERE GROUP 18 Hypertension
PitEnRRI =1.3543
JSDSh =5.0065
COP =0.15986
RR [ms]
Time [s]
SBP [mmHg]
Time [s]

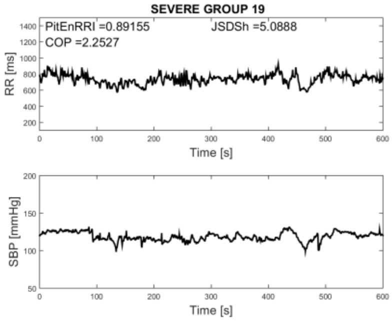
SEVERE GROUP 19
PitEnRRI =0.89155
JSDSh =5.0888
COP =2.2527
RR [ms]
Time [s]
SBP [mmHg]
Time [s]

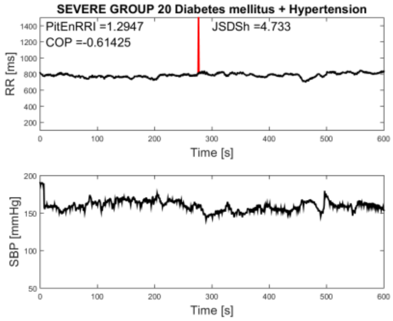
SEVERE GROUP 20 Diabetes mellitus + Hypertension
PitEnRRI =1.2947
JSDSh =4.733
COP =-0.61425
RR [ms]
Time [s]
SBP [mmHg]
Time [s]

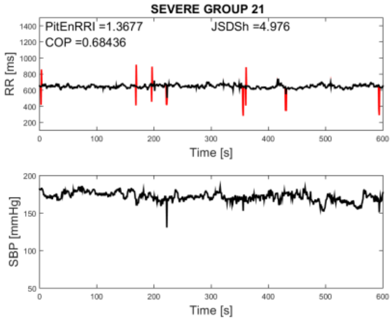
SEVERE GROUP 21
PitEnRRI =1.3677
JSDSh =4.976
COP =0.68436
RR [ms]
Time [s]
SBP [mmHg]
Time [s]

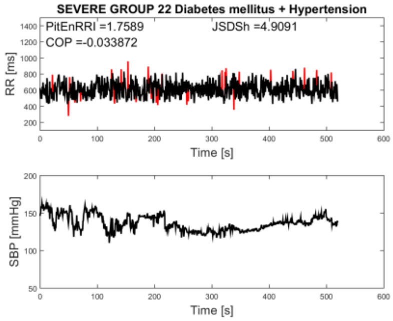
SEVERE GROUP 22 Diabetes mellitus + Hypertension
PitEnRRI =1.7589
JSDSh =4.9091
COP =-0.033872
RR [ms]
Time [s]
SBP [mmHg]
Time [s]

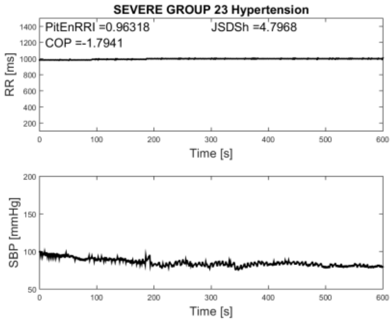
SEVERE GROUP 23 Hypertension
PitEnRRI =0.96318
JSDSh =4.7968
COP =-1.7941
RR [ms]
Time [s]
SBP [mmHg]
Time [s]

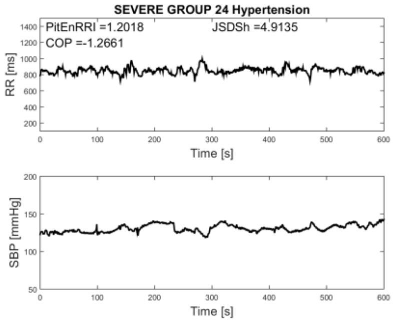
SEVERE GROUP 24 Hypertension
PitEnRRI =1.2018
JSDSh =4.9135
COP =-1.2661
RR [ms]
Time [s]
SBP [mmHg]
Time [s]

SEVERE GROUP 25

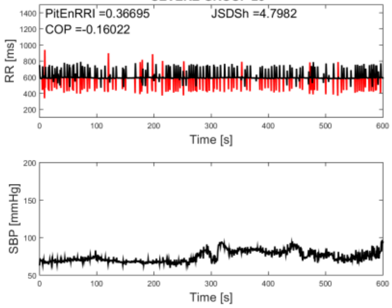

SEVERE GROUP 26

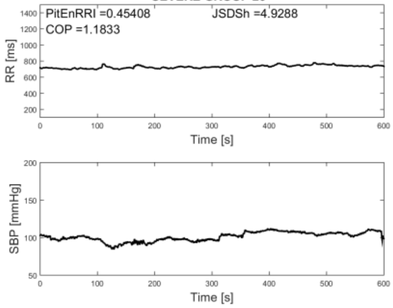

SEVERE GROUP 27 Syncope

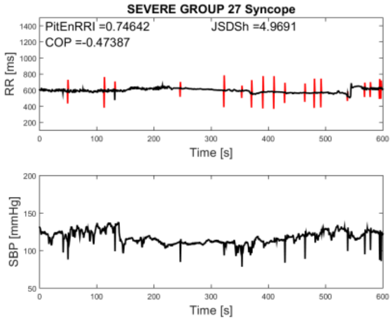

SEVERE GROUP 28

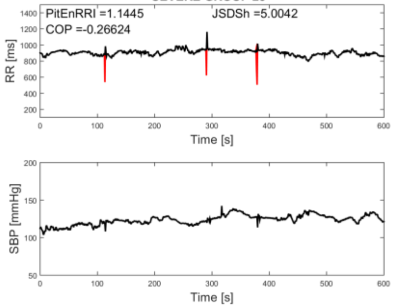

SEVERE GROUP 29

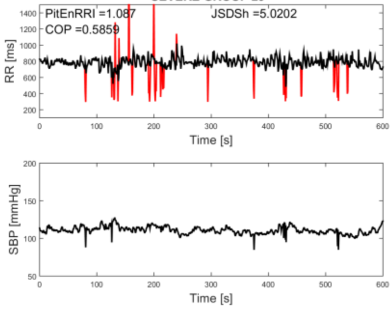

SEVERE GROUP 30 Syncope

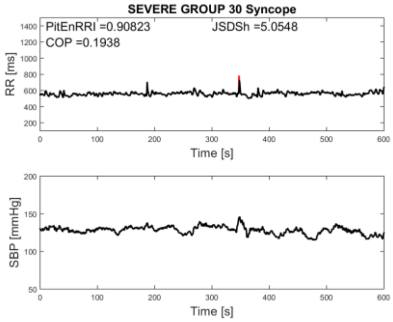

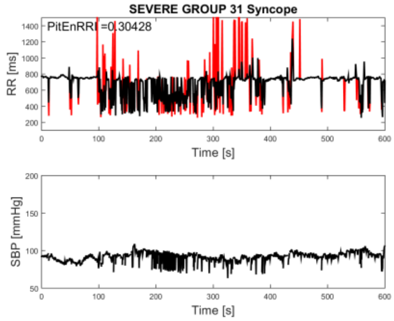
SEVERE GROUP 31 Syncope
PitEnRRI =0.30428
RR [ms]
Time [s]
SBP [mmHg]
Time [s]

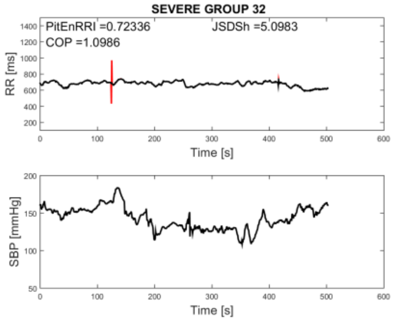
SEVERE GROUP 32
PitEnRRI =0.72336
JSDSh =5.0983
COP =1.0986
RR [ms]
Time [s]
SBP [mmHg]
Time [s]

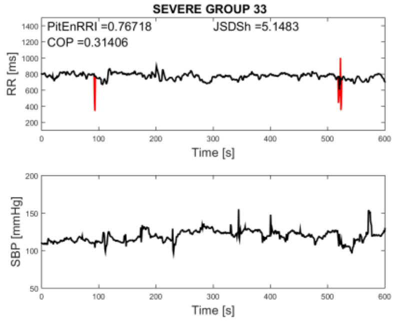
SEVERE GROUP 33
PitEnRRI =0.76718
JSDSh =5.1483
COP =0.31406
RR [ms]
Time [s]
SBP [mmHg]
Time [s]

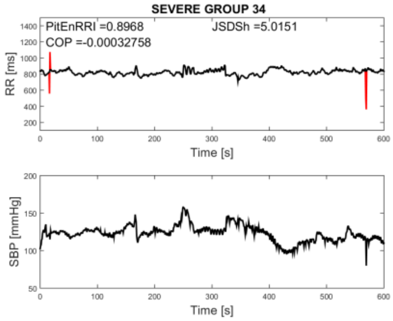
SEVERE GROUP 34
PitEnRRI =0.8968
JSDSh =5.0151
COP =-0.00032758
RR [ms]
Time [s]
SBP [mmHg]
Time [s]

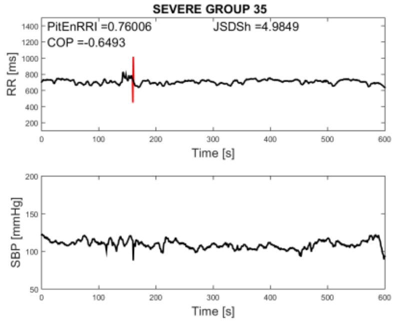
SEVERE GROUP 35
PitEnRRI =0.76006
JSDSh =4.9849
COP =-0.6493
RR [ms]
Time [s]
SBP [mmHg]
Time [s]

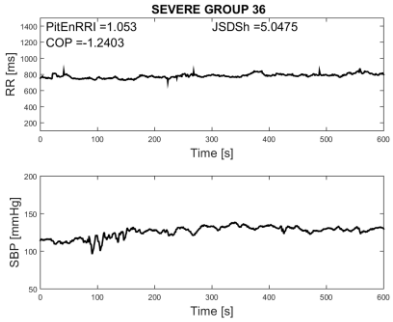
SEVERE GROUP 36
PitEnRRI =1.053
JSDSh =5.0475
COP =-1.2403
RR [ms]
Time [s]
SBP [mmHg]
Time [s]

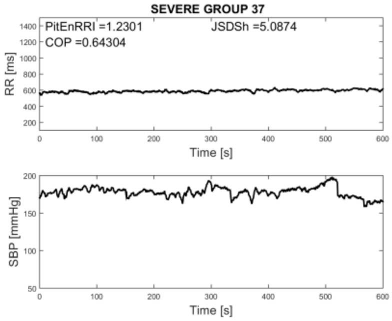
SEVERE GROUP 37
PitEnRRI =1.2301
COP =0.64304
JSDSh =5.0874
RR [ms]
Time [s]
SBP [mmHg]
Time [s]

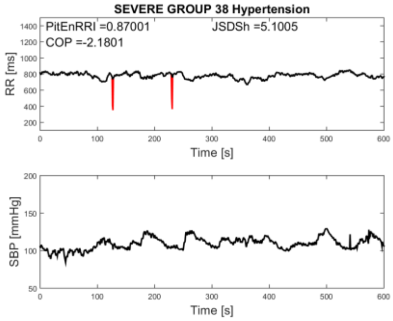
SEVERE GROUP 38 Hypertension
PitEnRRI =0.87001
COP =-2.1801
JSDSh =5.1005
RR [ms]
Time [s]
SBP [mmHg]
Time [s]

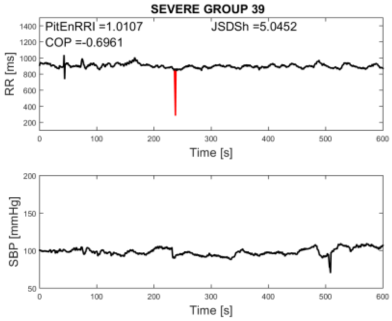
SEVERE GROUP 39
PitEnRRI =1.0107
COP =-0.6961
JSDSh =5.0452
RR [ms]
Time [s]
SBP [mmHg]
Time [s]

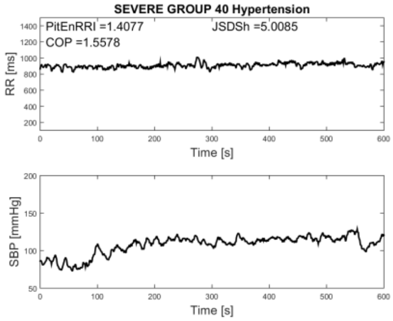
SEVERE GROUP 40 Hypertension
PitEnRRI =1.4077
COP =1.5578
JSDSh =5.0085
RR [ms]
Time [s]
SBP [mmHg]
Time [s]

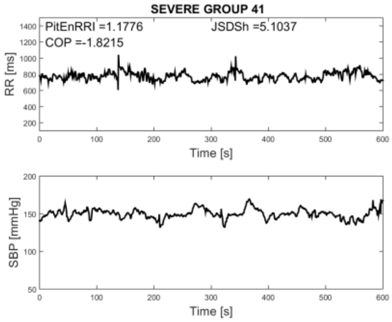
SEVERE GROUP 41
PitEnRRI =1.1776
COP =-1.8215
JSDSh =5.1037
RR [ms]
Time [s]
SBP [mmHg]
Time [s]

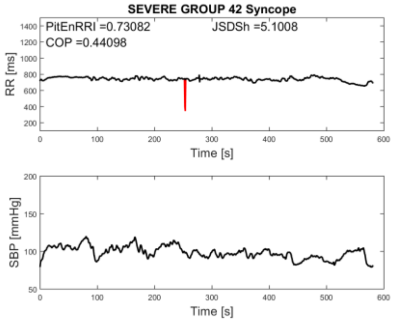
SEVERE GROUP 42 Syncope
PitEnRRI =0.73082
COP =0.44098
JSDSh =5.1008
RR [ms]
Time [s]
SBP [mmHg]
Time [s]

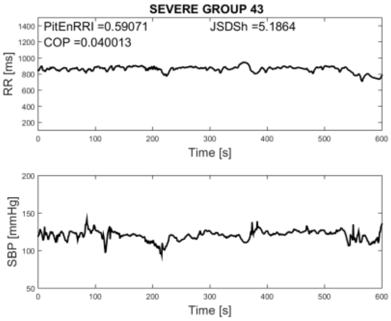
SEVERE GROUP 43
PitEnRRI =0.59071
JSDSh =5.1864
COP =0.040013
RR [ms]
Time [s]
SBP [mmHg]
Time [s]

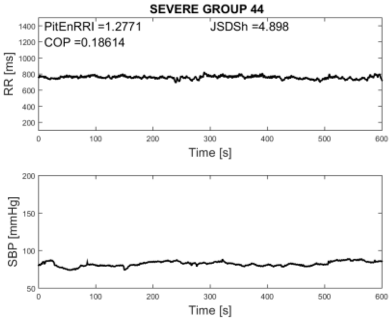
SEVERE GROUP 44
PitEnRRI =1.2771
JSDSh =4.898
COP =0.18614
RR [ms]
Time [s]
SBP [mmHg]
Time [s]

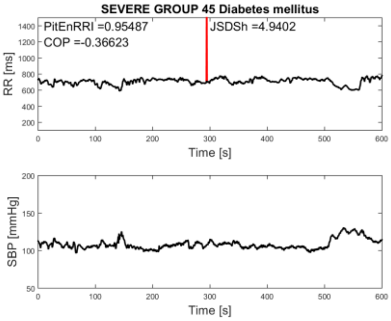
SEVERE GROUP 45 Diabetes mellitus
PitEnRRI =0.95487
JSDSh =4.9402
COP =-0.36623
RR [ms]
Time [s]
SBP [mmHg]
Time [s]

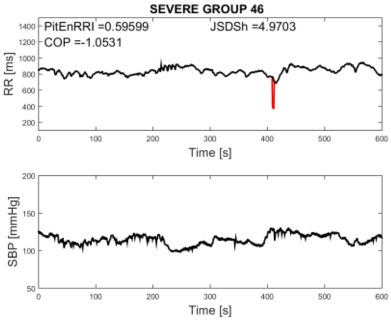
SEVERE GROUP 46
PitEnRRI =0.59599
JSDSh =4.9703
COP =-1.0531
RR [ms]
Time [s]
SBP [mmHg]
Time [s]

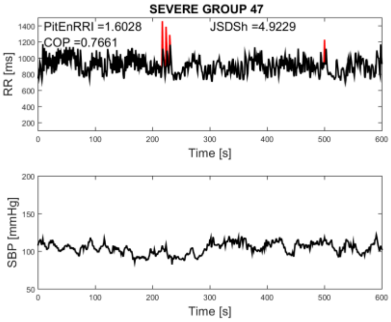
SEVERE GROUP 47
PitEnRRI =1.6028
JSDSh =4.9229
COP =0.7661
RR [ms]
Time [s]
SBP [mmHg]
Time [s]

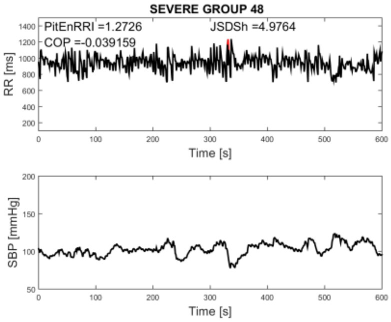
SEVERE GROUP 48
PitEnRRI =1.2726
JSDSh =4.9764
COP =-0.039159
RR [ms]
Time [s]
SBP [mmHg]
Time [s]

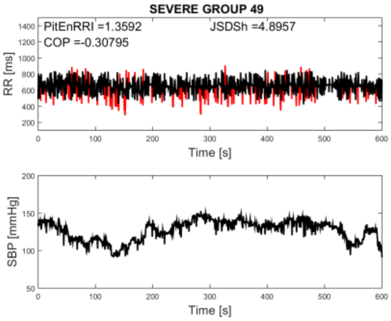
SEVERE GROUP 49
PitEnRRI =1.3592
JSDSh =4.8957
COP =-0.30795
RR [ms]
Time [s]
SBP [mmHg]
Time [s]

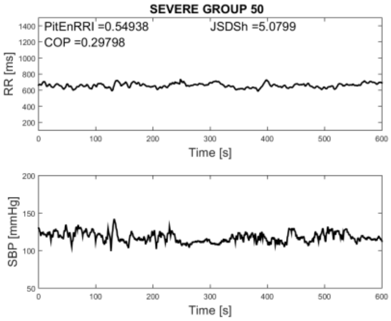
SEVERE GROUP 50
PitEnRRI =0.54938
JSDSh =5.0799
COP =0.29798
RR [ms]
Time [s]
SBP [mmHg]
Time [s]

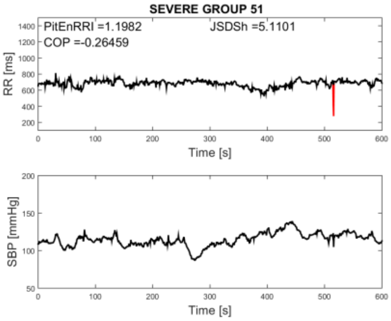
SEVERE GROUP 51
PitEnRRI =1.1982
JSDSh =5.1101
COP =-0.26459
RR [ms]
Time [s]
SBP [mmHg]
Time [s]

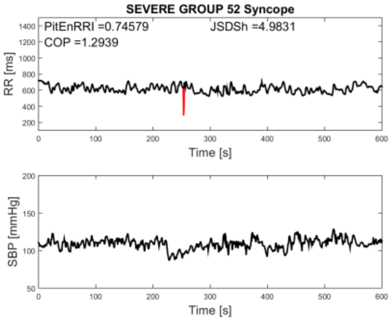
SEVERE GROUP 52 Syncope
PitEnRRI =0.74579
JSDSh =4.9831
COP =1.2939
RR [ms]
Time [s]
SBP [mmHg]
Time [s]

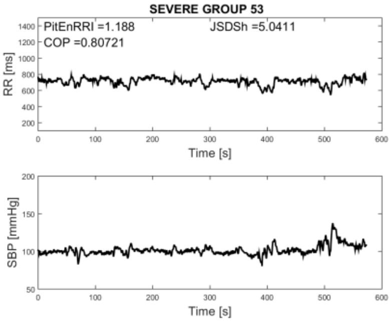
SEVERE GROUP 53
PitEnRRI =1.188
JSDSh =5.0411
COP =0.80721
RR [ms]
Time [s]
SBP [mmHg]
Time [s]

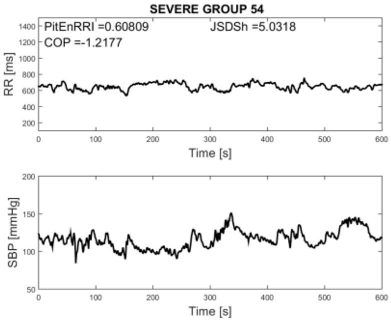
SEVERE GROUP 54
PitEnRRI =0.60809
JSDSh =5.0318
COP =-1.2177
RR [ms]
Time [s]
SBP [mmHg]
Time [s]

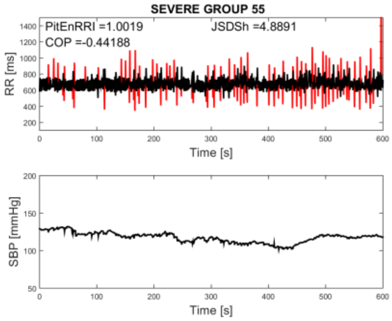
SEVERE GROUP 55
PitEnRRI =1.0019
JSDSh =4.8891
COP =-0.44188
RR [ms]
Time [s]
SBP [mmHg]
Time [s]

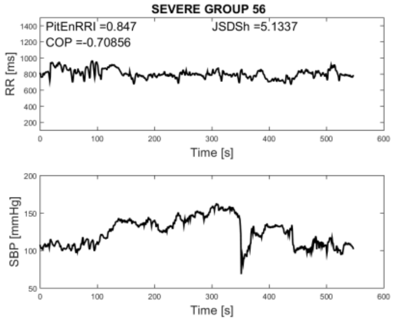
SEVERE GROUP 56
PitEnRRI =0.847
JSDSh =5.1337
COP =-0.70856
RR [ms]
Time [s]
SBP [mmHg]
Time [s]

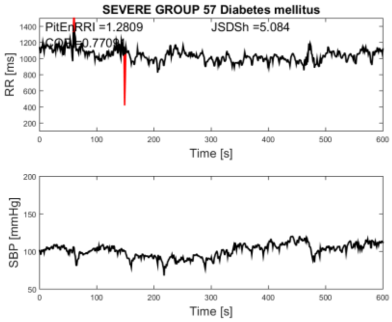
SEVERE GROUP 57 Diabetes mellitus
PitEnRRI =1.2809
JSDSh =5.084
RR [ms]
Time [s]
SBP [mmHg]
Time [s]

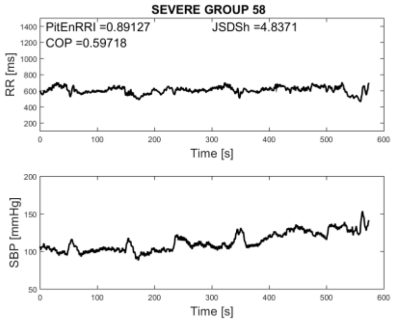
SEVERE GROUP 58
PitEnRRI =0.89127
JSDSh =4.8371
COP =0.59718
RR [ms]
Time [s]
SBP [mmHg]
Time [s]

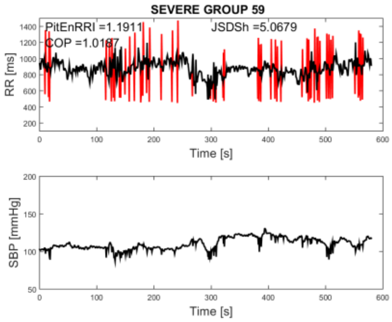
SEVERE GROUP 59
PitEnRRI =1.1911
JSDSh =5.0679
RR [ms]
Time [s]
SBP [mmHg]
Time [s]

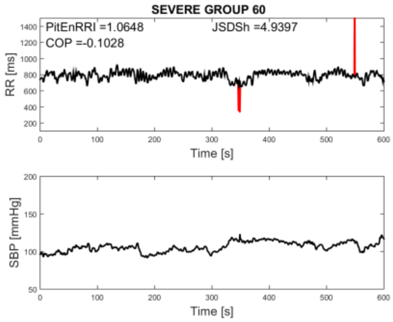
SEVERE GROUP 60
PitEnRRI =1.0648
JSDSh =4.9397
COP =-0.1028
RR [ms]
Time [s]
SBP [mmHg]
Time [s]

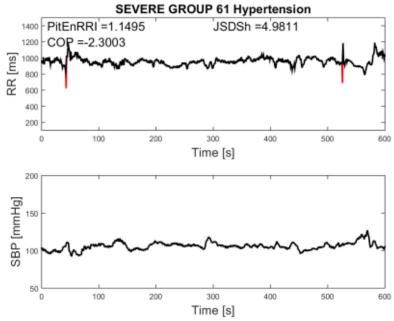
SEVERE GROUP 61 Hypertension
PitEnRRI =1.1495
JSDSh =4.9811
COP =-2.3003
RR [ms]
Time [s]
SBP [mmHg]
Time [s]

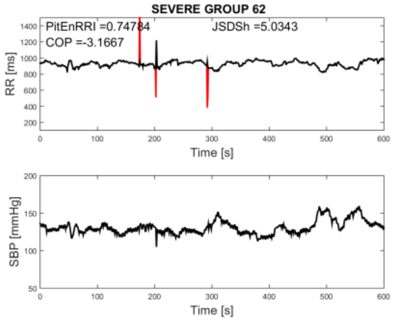
SEVERE GROUP 62
PitEnRRI =0.74784
JSDSh =5.0343
COP =-3.1667
RR [ms]
Time [s]
SBP [mmHg]
Time [s]

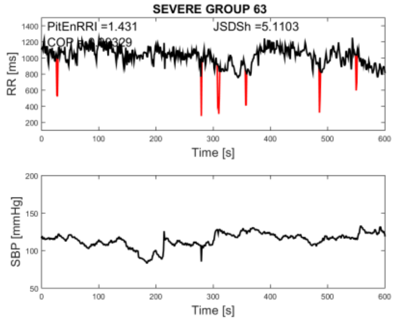
SEVERE GROUP 63
PitEnRRI =1.431
JSDSh =5.1103
RR [ms]
Time [s]
SBP [mmHg]
Time [s]

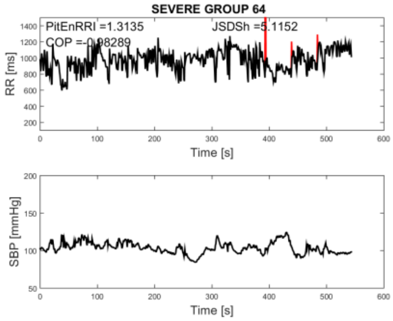
SEVERE GROUP 64
PitEnRRI =1.3135
JSDSh =5.1152
COP =-0.98289
RR [ms]
Time [s]
SBP [mmHg]
Time [s]

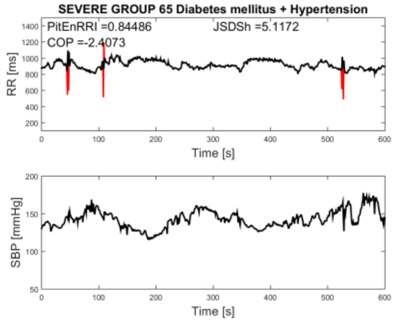
SEVERE GROUP 65 Diabetes mellitus + Hypertension
PitEnRRI =0.84486
JSDSh =5.1172
COP =-2.4073
RR [ms]
Time [s]
SBP [mmHg]
Time [s]

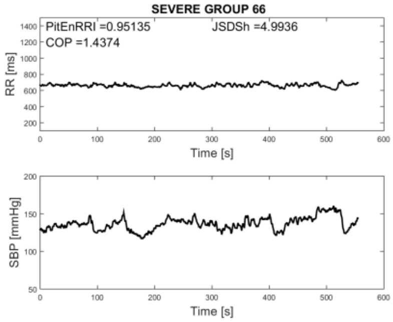
SEVERE GROUP 66
PitEnRRI =0.95135
JSDSh =4.9936
COP =1.4374
RR [ms]
Time [s]
SBP [mmHg]
Time [s]

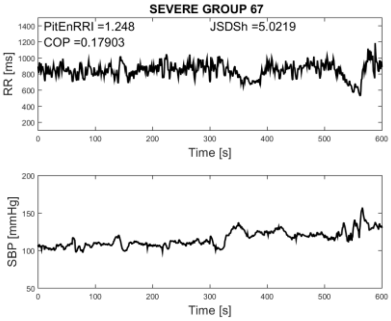
SEVERE GROUP 67
PitEnRRI =1.248
COP =0.17903
JSDSh =5.0219
RR [ms]
Time [s]
SBP [mmHg]
Time [s]

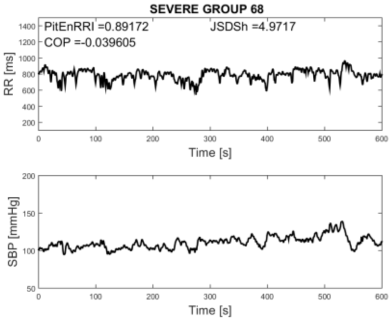
SEVERE GROUP 68
PitEnRRI =0.89172
COP =-0.039605
JSDSh =4.9717
RR [ms]
Time [s]
SBP [mmHg]
Time [s]

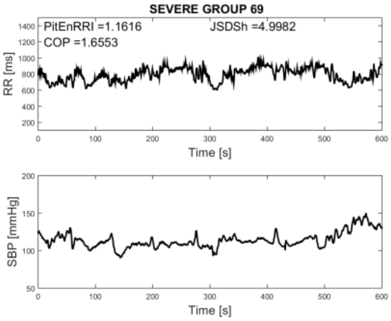
SEVERE GROUP 69
PitEnRRI =1.1616
COP =1.6553
JSDSh =4.9982
RR [ms]
Time [s]
SBP [mmHg]
Time [s]

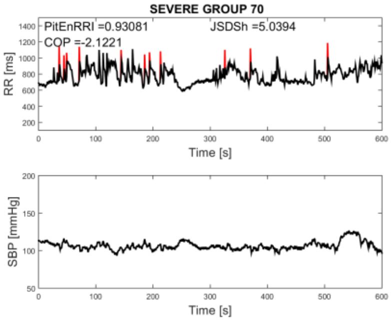
SEVERE GROUP 70
PitEnRRI =0.93081
COP =-2.1221
JSDSh =5.0394
RR [ms]
Time [s]
SBP [mmHg]
Time [s]
